# *De novo* transcriptome assembly of the Chinese pearl barley, adlay, by full-length isoform and short-read RNA sequencing

**DOI:** 10.1371/journal.pone.0208344

**Published:** 2018-12-11

**Authors:** Sang-Ho Kang, Jong-Yeol Lee, Tae-Ho Lee, Soo-Yun Park, Chang-Kug Kim

**Affiliations:** 1 International Technology Cooperation Center, RDA, Jeonju, Republic of Korea; 2 Metabolic Engineering Division, National Institute of Agricultural Sciences, RDA, Jeonju, Korea; 3 Genomics Division, National Institute of Agricultural Sciences, RDA, Jeonju, Korea; 4 Biosafety Division, National Institute of Agricultural Sciences, RDA, Jeonju, Korea; ICAR-Indian Institute of Agricultural Biotechnology, INDIA

## Abstract

Adlay (*Coix lacryma-jobi*) is a tropical grass that has long been used in traditional Chinese medicine and is known for its nutritional benefits. Recent studies have shown that vitamin E compounds in adlay protect against chronic diseases such as cancer and heart disease. However, the molecular basis of adlay's health benefits remains unknown. Here, we generated adlay gene sets by *de novo* transcriptome assembly using long-read isoform sequencing (Iso-Seq) and short-read RNA-Sequencing (RNA-Seq). The gene sets obtained from Iso-seq and RNA-seq contained 31,177 genes and 57,901 genes, respectively. We confirmed the validity of the assembled gene sets by experimentally analyzing the levels of prolamin and vitamin E biosynthesis-associated proteins in adlay plant tissues and seeds. We compared the screened adlay genes with known gene families from closely related plant species, such as rice, sorghum and maize. We also identified tissue-specific genes from the adlay leaf, root, and young and mature seed, and experimentally validated the differential expression of 12 randomly-selected genes. Our study of the adlay transcriptome will provide a valuable resource for genetic studies that can enhance adlay breeding programs in the future.

## Introduction

Adlay (*Coix lacryma-jobi*), also called as Job’s tears or Chinese pearl barley, is a panicoid grass that belongs to the Poaceae family. This annual crop is widely grown in China, Japan, Taiwan, Thailand, and Korea, where it is a popular nutritional supplement used in traditional medicine. Adlay has been used to treat warts, chapped skin, neuralgia, and rheumatism. Adlay seeds have exhibited anti-inflammatory[1–4], anti-mutagenic [5], anti-cancer [6–12], anti-allergic [13–15], anti-obesity [16–19], anti-microbial [20], and anti-oxidant [21–24] properties.

The adlay seed contains large amounts of storage proteins called prolamins [25]. Prolamins are known as zeins in maize, kafirins in sorghum, coixins in adlay, and caneins in sugarcane. In adlay, prolamins are grouped into two subclasses, α- and γ-coixins. The dominant storage protein, α-coixin, consists of two size classes with molecular weights of 19 and 22 kDa[26–29]. Adlay is widely used as a nourishing food and is enriched in valuable compounds such as vitamin E, squalene, and phytosterols [30]. Vitamin E, a potent anti-oxidant, consists of eight major isoforms: four saturated tocopherols (α, β, γ, and δ) and four unsaturated tocotrienols (α, β, γ, and δ) [29, 30]. In recent studies, adlay has been shown to protect against cardiovascular disease [31, 32], cancer [33, 34], and other chronic diseases [35–41].

Gene expression studies help select significant candidate genes for transcriptome profiling to gain better understanding of cellular pathways and metabolism in plants [42]. Genome-wide transcriptome studies for adlay are currently lacking. In this study, we combined Illumina short-read sequencing with single-molecule real-time (SMRT) sequencing to generate a complete and full-length transcriptome of adlay. Transcriptome analysis revealed several tissue-specific and stage-specific gene expression patterns. Comparative analysis of gene families between species revealed that adlay was closest in relation to sorghum. Our comprehensive transcriptome data set provides a valuable resource for further genetic research on adlay,

## Materials and methods

### Plant material and RNA preparation

*Coix lacryma-jobi* L. (voucher # IT101241, var. *ma-yuen* Stapf) was obtained from the National Institute of Agricultural Sciences (NAS, Wanju, Korea). Ninety-eight-day-old leaves, roots, and young seeds and 159-day-old mature seeds were harvested from adlay plants and stored at -80°C until used. RNA-sequencing (RNA-Seq) and isoform sequencing (Iso-Seq) were performed on leaf, root, young seed, and mature seed tissues (S1 Fig). Three biological replicates were used for RNA-seq. Total RNA was extracted from leaf, root, and the two seed stages (young and mature) using RNeasy Plant Mini kit (Qiagen, Inc., USA). RNA purity was determined by assaying 1 μl total RNA on a NanoDrop8000 Spectrophotometer (ThermoFisher, USA). Total RNA integrity was checked using an Agilent Technologies 2100 Bioanalyzer with minimum integrity option.

### Long-read sequencing for Iso-Seq

To produce full-length isoforms, long-read sequencing was performed using Pacific Biosciences (PacBio, USA) SMRT sequencing. Libraries were prepared from cDNAs, and cycle optimization was performed to determine the optimal number of cycles for large-scale PCR. We prepared three fractions of cDNAs (1–2 Kbp, 2–3 Kbp, and 3–6 Kbp) using the BluePippin size selection system (Sage Science, USA). The SMRTbell library was constructed using the SMRTbell^™^ Template Prep Kit (PN 100-259-100; PacBio). The DNA/Polymerase Binding Kit P6 (PacBio) was used for DNA synthesis after the sequencing primer annealed to the SMRTbell template. MagBead Kit (PacBio) was used to attach the cDNA library to MagBeads before sequencing. MagBead-bound cDNA complexes result in increased number of reads per SMRT cell. The polymerase-SMRTbell-adaptor complex was then loaded into zero-mode waveguides (ZMWs). The SMRTbell library was sequenced using 8 SMRT cells using C4 chemistry (DNA sequencing Reagent 4.0; PacBio) and 1 × 240-minute movies were captured for each SMRT cell using the PacBio RS II sequencing platform.

### Short-read sequencing for RNA-seq

The poly(A)^+^ mRNA was purified and fragmented from 1 μg total RNA using poly-T oligo-attached magnetic beads after two rounds of purification. Cleaved RNA fragments primed with random hexamers were reverse transcribed into first-strand cDNAs using reverse transcriptase, random hexamer primers, and dUTPs in place of dTTPs. A single A-base was added to the cDNA fragments and the adapter was subsequently ligated to the cDNA. The products were purified and enriched by polymerase chain reaction (PCR) to create the final strand-specific cDNA library. The quality of the amplified libraries was verified by capillary electrophoresis using Bioanalyzer 2100 (Agilent, Germany). Quantitative PCR (qPCR) was performed using SYBR Green PCR Master Mix (Applied Biosystems, ThermoFisher, USA). We pooled together equimolar amounts of libraries that were index-tagged. The cBot2 system was used for automated cluster generation in the flow cell. (Illumina Inc., USA). The flow cell was loaded on HiSeq 2500 sequencing system (Illumina Inc.) and cDNA was sequenced at a read length of 2 × 100 bp.

### *De novo* transcriptome assembly

All transcriptome data were processed using next generation sequencing quality control toolkit [43] to remove nonsense sequences such as adaptors, primers, and low quality sequences (Phred quality score of less than 20). Raw data were processed to remove ribosomal RNA using riboPicker v0.4.3 [44]. The processed reads were then assembled using Trinity [45]. Assembly statistics were calculated using in-house Perl scripts. Assembled transcripts were clustered using CD-HIT-EST v4.6.1 [46] to reduce sequence redundancy. Sequence identity threshold and alignment coverage (for shorter sequences) were both set at 90% to generate clusters. Such clustered transcripts were defined as reference transcripts in this work. For functional annotation, RNA-Seq unigenes were screened using a fragments per kilobase of transcript per million mapped reads (FPKM) criterion of ≥ 1 for each unigene, and non-redundant Iso-Seq unigens were screened using different libraries (<2, 2–3, and >3 kb) with considerably larger average lengths (≥340 bp).

### Functional annotation

All the assembled genes were annotated by the Basic Local Alignment Search Tool (BLAST) program [47] using databases such as NCBI non-redundant protein database (NR, https://www.ncbi.nlm.nih.gov/), UniProtKB/Swiss-Prot (Swissprot, https://web.expasy.org/), Kyoto Encyclopedia of Genes and Genomes (KEGG, http://www.genome.jp/kegg/), and Gene Ontology Consortium (GO, http://www.geneontology.org/) with an Expect Value (E-value) cutoff of 10^−5^. The best-aligned sequences were selected to annotate genes. In the event of alignment conflicts between databases, Swissprot alignments were preferentially selected. For functional categorization, GO terms were produced by Blast2GO program [48] with an E-value threshold of 10^−5^ and GO terms were classified into three major categories using the Web Gene Ontology Annotation Plot (WEGO) [49].

### Gene expression analysis

Expressed genes from each tissue were aligned with the adlay transcriptome assembly using Bowtie2 [50]. Genes that aligned with non-redundant assembled transcript sequences were quantified as FPKMs (at 90% sequence similarity by CD-HIT-EST). Quantification was performed using RNA-Seq by Expectation Maximization v1.2.25 (RSEM) [51]. Differential expression analysis was performed using DESeq2 packages (Bioconductor) [52]. Differentially expressed genes during seed development (young and mature seed stages) were screened from three biological replicate experiments based on log fold change values > 1 (p values < 0.001). For specificity classification, each tissue/organ was classified into two categories based on the FPKM values. A ten-fold increase in FPKM values was used as a criterion for tissue specificity and a five-fold increase was used as a criterion for tissue enrichment. ClueGo plug-in tool [53] in Cytoscape v3.3.0 was used to identify over- representation of GO categories, such as biological processes. We also screened for potential transcription factor families in the Plant TF Database [54] (PlantTFDB 4.0; http://planttfdb.cbi.pku.edu) using BLASTX with an E-value threshold of 10^−5^.

### Gene family identification

OrthoMCL [55] (version 2.0.3) was used to identify orthologous and paralogous gene clusters of adlay with three other members of the Poaceae family, rice (*O*. *sativa*, NCBI accession number GCF_001433935), sorghum (*S*. *bicolor*, GCF_000003195), and maize (*Z*. *mays*, GCF_000005005). Protein sequences that were shorter than 10 amino acids or contained >20% stop codons were removed. All-versus-all analysis was performed with BLASTP version 2.2.25+ (E value threshold 10^−5^). The protein pairs obtained from BLASTP analysis were processed using the OrthoMCL program (http://orthomcl.org/orthomcl/).

### Quantitative real-time PCR analysis

The quality of RNA isolated from *C*. *lacryma-jobi* was checked on ethidium bromide-stained agarose gels, and RNA concentration was calculated based on the measured optical density of the samples at 260 and 280 nm using a DropSense96C Spectrophotometer (Trinean, Belgium). One microgram of total RNA was used for cDNA synthesis, which was performed using a SuperScript^™^ III first strand RT-PCR kit (Invitrogen, USA) with an oligo (dT)_20_ primer. Quantitative real-time PCR (qRT-PCR) was performed on synthesized cDNA using gene-specific primers (S1 Table). PCR was optimized and performed using the Roche LightCycler 480 II and SYBR Green Real-Time PCR Master Mix (Bio-Rad,Inc., CA). Reaction conditions included an initial denaturation at 95°C for 30 s, 40 cycles of denaturation at 95°C for 10 s, and annealing and extending at 55°C for 15 s. The relative expressions of specific genes were quantified using the 2^-ΔΔCt^ method [56].

### Verification and identification of prolamin-coding genes

Genes that encoded for prolamin proteins (coixins in *Coix lacryma-jobi*) were analyzed by BLAST using the NCBI non-redundant database. Longest open reading frames (ORFs) and amino acid sequences of selected genes that showed similarity to prolamins were predicted by TransDecoder (https://github.com/TransDecoder/TransDecoder/wiki). The theoretical isoelectric point (pI) and molecular weight of the predicted protein sequences were calculated using the pI/Mw computing tool in ExPASy (https://web.expasy.org/compute_pi/). For phylogenetic analysis, multiple sequence alignments of the predicted amino acid sequences were performed using MUSCLE tool (MEGA 7 software; https://www.megasoftware.net/). Based on the alignments, the phylogenetic tree was generated using the Neighbor-joining method (MEGA 7) with the following parameters: Poisson model, pair-wise gap deletion and 1,000 bootstraps. Protein sequences of the previously reported 31 prolamin genes in *C*. *lacryma-jobi* [28, 29] were also included in the tree for comparison.

Adlay prolamin was extracted based by the method described in a previous study [29]. Young and mature seeds were finely pulverized using liquid nitrogen. Hundred milligrams of pulverized adlay seeds were mixed with 1 ml of 0.5 M NaCl at room temperature for 1 hour and centrifuged at 4°C, 12,000 × g for 5 minutes. The supernatant was removed and the pellet containing prolamin was washed three times with cold, sterilized water and dried at room temperature. The pellet was mixed with 500 μl of 10 mM 1,4-dithiothreitol (DTT) in 55% v/v isopropanol for 1 hour at room temperature. After centrifugation, 200 μl supernatant and 800 μl cold acetone were mixed and stored at -20°C for more than 3 hours to precipitate prolamin. For two-dimensional gel electrophoresis (2-DGE), the precipitated prolamin was centrifuged at 12,000 × g for 5 minutes at 4°C and the supernatant was removed. The pellet was mixed with 50 μl rehydration buffer (7 M urea, 2 M thiourea, 2% CHAPS detergent, 0.5% IPG buffer) containing 1 M DTT and prolamin was quantified using the Bradford protein assay [57]. The rehydration buffer (350 μl) containing 110 mg of prolamin was loaded onto an immobilized pH gradient (IPG) strip (pI 6–11, 18cm, GE Healthcare Life Sciences, USA). The IPG strip was loaded into the IPGphor system (GE Healthcare Life Sciences) for 25 hours for isoelectric focusing. Subsequently, the IPG strip was placed on a 15% sodium dodecyl sulphate polyacrylamide (SDS-PAGE) gel and 2D-gel electrophoresis was performed for 23 hours. The gel was stained by Coomassie Brilliant Blue R250 for 3 hours and de-stained for 3 hours. Gels were analyzed and spot volumes were measured using Image Master Platinum 6.0 (GE Healthcare Life Sciences).

### Verification and identification of vitamin E-coding genes

Genes that encoded for vitamin E-related proteins were screened by BLAST using 31,177 genes in the NCBI non-redundant database. A heat map was generated using DNAStar's ArrayStar 4 (http://www.dnastar.com). Plant samples were lyophilized at -80°C for 4 days and pulverized into a very fine powder using a planetary mono mill (Pulverisette 6; Fritsch, Germany). Tocopherols and tocotrienols were identified by gas chromatography-time-of-flight mass spectrometry according to a previously described method [58]. Lipophilic compounds were extracted from 0.1 g of samples by adding 3 ml of ethanol containing 0.1% ascorbic acid (w/v); 0.05 ml of 5 α-cholestane (10 μg/mL) was used as an internal standard (IS). The extracts were lyophilized and then derivatized with 30 μl *N*-methyl-*N*-trimethylsilyltrifluoro-actamide (Sigma, USA) and 30 μl pyridine. The derivatized extracts were analyzed by a 7890A gas chromatograph (Agilent, USA) with a Pegasus HT TOF mass spectrometer (LECO, USA). Tocopherols and tocotrienols were quantified using calibration curves that plotted five concentrations of the commercial standards ranging from 0.01 to 10.0 μg and a fixed amount (0.5 μg each) of IS.

## Results and discussion

### *De novo* assembly of the adlay transcriptome

We performed RNA-Seq and Iso-Seq on tissues obtained from adlay leaf, root, and young and mature seeds. RNA-Seq data were obtained from 17.3 million to 21.5 million reads per tissue sample. In total, more than 230 million reads showed high quality read rates (Q30 values) of over 90% (S2 Table). The high-quality reads were assembled *de novo* using the Trinity assembler, which generated 111,850 unigenes that were more than 300 basepairs (bp) long. The contigs, after removal of redundant transcripts by the CD-HIT-EST program [46] were distributed as follows: N10 (3,675), N20 (2,760), N30 (2,203), N40 (1,761), and N50 (1,367).

A unigene is a uniquely assembled transcript that denotes a hypothetical gene, which may be represented by multiple isoforms as several different forms of the same protein. Iso-Seq data produced 110,645 high-quality isoforms from three different libraries (S3 Table), which generated 31,177 non-redundant unigenes that were more than 300 bp in length. In total, our analysis generated two unigene sets: 111,850 from RNA-Seq and 31,177 from Iso-Seq. Comparison of the two unigene sets revealed similar GC contents (S4 Table). However, the distribution ratio of unigenes was higher for Iso-Seq compared with RNA-seq with increasing unigene lengths (S2 Fig). Although the number of unigenes obtained from RNA-Seq and their total size was higher for RNA-seq, unigenes obtained from Iso-Seq were better in terms of minimum length, average length, and N50 length (S4 Table). Similar results have been reported for *Salvia miltiorrhiza* [59].

In our subsequent analyses, we used the Iso-Seq unigene set mainly as a reference for RNA-Seq data. We did not construct an integrated unigene set in this study because of dissimilar characteristics between RNA-Seq and Iso-Seq gene sets, such as transcript lengths and mRNA abundance. We plan to create the integrated gene set using the reference-guided method when the adlay genome sequencing is completed.

### Functional annotation

For functional annotation, the assembled 111,850 unigenes obtained from RNA-Seq of leaf, root, young seed, and mature seed tissue samples were screened using an FPKM criterion of ≥ 1, which resulted in 57,901 unigenes. The RNA-Seq unigene set of 57,901 and the Iso-Seq unigene set of 31,177 were annotated using BLAST searches against NCBI/NR, Swissprot, KEGG, and GO databases. The annotated RNA-Seq genes were distributed in NR (59.4%), Swissprot (42.4%), KEGG (17.3%), and GO (36.6%) with duplicates. A total of 60.6% of genes showed significant protein matches in at least one of the four databases (S3A Fig). The annotated Iso-Seq genes were distributed in NR (90.8%), Swissprot (72.5%), KEGG (15.0%), and GO (60.4%). A total of 91.3% of genes showed significant protein matches in at least one of these databases (S3B Fig). The annotation patterns showed that the Iso-Seq unigene set contained more full-length genes than the RNA-Seq unigene set. Non-significant genes that may represent novel genes, long non-coding RNAs, or less conserved 5’ or 3’ untranslated regions [60, 61] were not evaluated in this annotation and require further analysis.

For further functional categorization, GO terms were classified into three major categories: biological process, molecular function, and cellular components (S4 Fig). In the cellular components category, most genes were assigned to the cell and cell part sub-categories. In the molecular function category, the predominant sub-categories were binding and catalytic and in the biological process category, the predominant sub-categories were cellular process and metabolic process. This gene distribution pattern was similar to that seen in other medicinal plants [62, 63]. Of the 78 transcription factors that were screened, we found the most number of genes assigned to three transcription factors, MYB, bHLH, and AP2-EREBP (S5 Fig). BLAST analysis of adlay transcripts with different species revealed that adlay's sequences mostly matched the sequences of *Sorghum bicolor* (S6 Fig). This relationship has been previously observed during analysis of specialized metabolic pathways and repetitive sequences [64, 65].

### Gene expression in RNA-Seq

**Fig 1:**
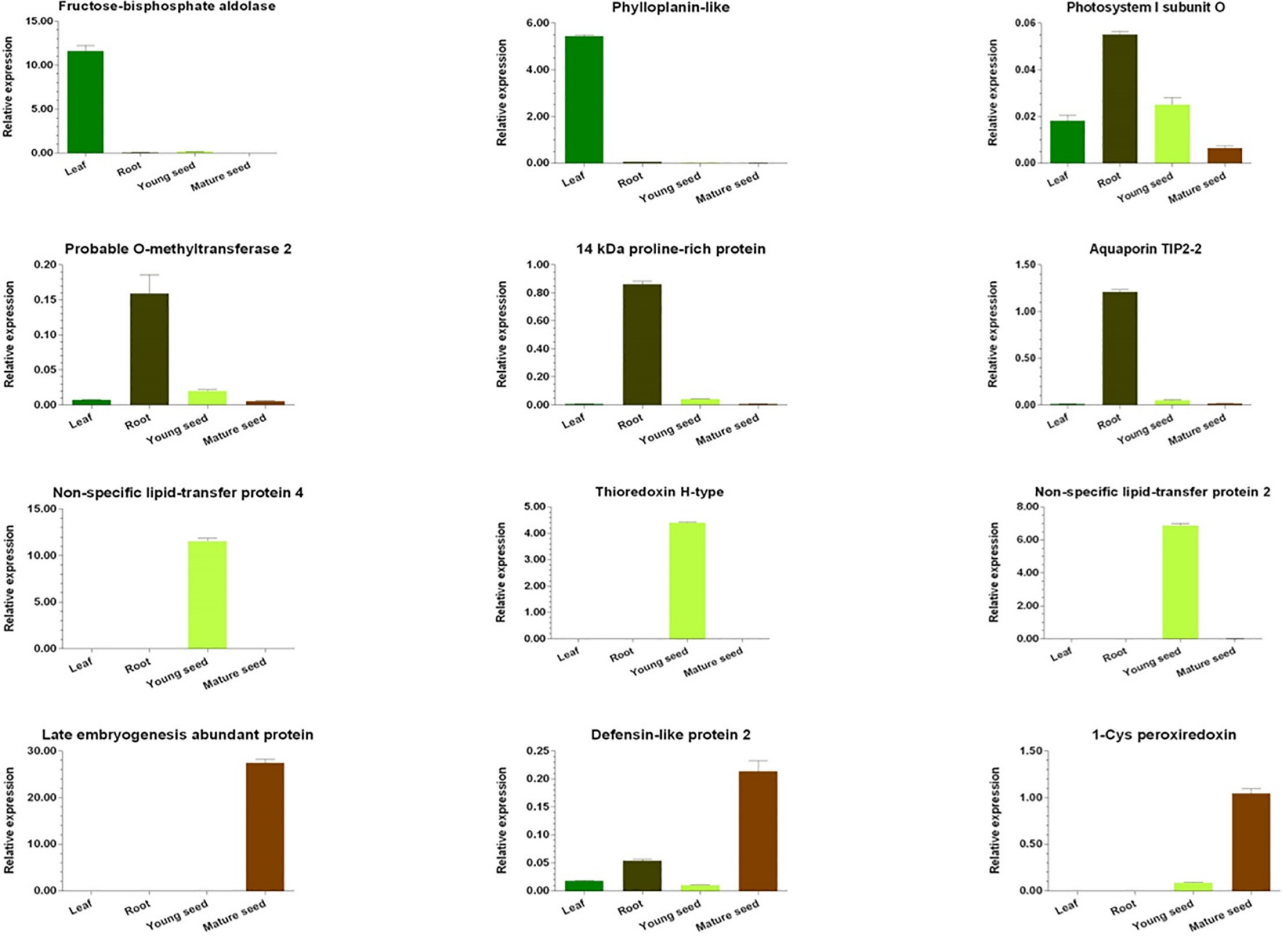
Analysis of tissue/organ-specific gene expression. Total RNA was extracted from leaf, root, and young and mature seed tissues and the expression levels of 12 genes were analyzed by qRT-PCR.

To investigate tissue-specific gene expression in adlay plants, we studied the expression of genes in leaf, root, and young and mature seed tissues. Based on three experimental replicates, we classified the expressed genes into two categories, tissue-specific and tissue-enriched (S5 Table). Ten tissue-specific genes were identified in each of the four tissues (S7 Fig). To validate tissue-specific genes, we randomly selected three genes from each tissue to perform qRT-PCR. The results were consistent with our tissue-specific gene expression data except for photosystem I subunit and defensin-like protein, which appeared to show expression in multiple tissues (Fig 1).

To identify the putative biological processes involved during seed ripening, we selected 25 genes that showed the highest upregulation and downregulation in expression during each of the seed development stages (S8 Fig). GO studies identified eight biological processes from the up-regulated gene set and 32 biological processes from the down-regulated gene set during the seed development process (S9 Fig). Up-regulated genes were prominent in organic biosynthetic processes or amino acid metabolic processes and a high number of down-regulated genes were seen in photosynthesis-related metabolic processes. We identified three heat shock-related proteins (HSPs) and two late embryogenesis-abundant (LEA) proteins that were increased in relative transcript abundance in mature seeds compared with young seeds. This may be the reason for increased desiccation and stress tolerance seen in mature seeds, which is consistent with previous studies that have shown a role for LEA and HSP proteins in stress tolerance in plants [66, 67]. Amino acid metabolism has been shown to be the major pathway during the kernel development in maize [68]. Our results show an increased number of putative genes involved in amino acid metabolism during seed maturation in adlay. Up-regulated unigenes were largely involved in organic or amino acid metabolic (or biosynthetic) processes and downregulated unigenes were mostly involved in photosynthesis-related metabolic (or biosynthetic) processes. Therefore, mature adlay seeds may be rich in amino acids, which may explain why they have long been used as a nourishing food for humans. Our study showed that RNA-Seq is a valuable tool to understand tissue-specific pathways in plants.

**Fig 2:**
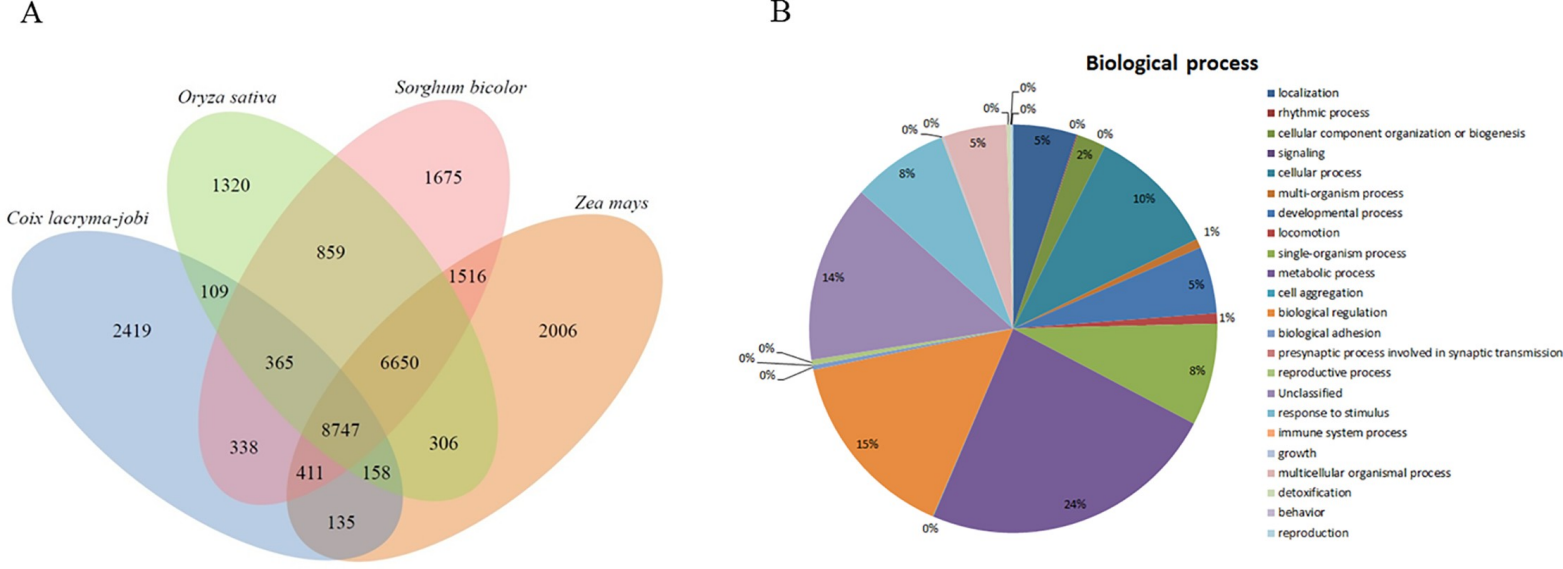
The distribution of 27,014 gene families among four monocot genomes. (A) Shared and unique gene families in *C*. *lacryma-jobi*, *O*. *sativa*, *S*. *bicolor*, and *Z*. *mays*. (B) GO analysis of the distribution of unique gene families of *C*. *lacryma-jobi* in various biological processes.

To study the relationship between adlay and other plant species, we categorized adlay transcripts into gene families and compared them with gene families from three other monocot plant species, rice (*O*. *sativa*), sorghum (*S*. *bicolor*), and maize (*Z*. *mays*). We found that a total of 8,747 gene families were shared by all four species, but 2,419 gene families were specific to adlay (Fig 2A). These adlay-specific genes included transposons, transposon elements, 19KDa alpha coixin-like, and alpha-coixin family of proteins. Using the 2,419 adlay-specific genes, we showed that adlay's sequences matched the sequences of *Sorghum bicolor* (49%), *Zea mays* (35%), and *Oryza sativa* (12%). Therefore, adlay was most closely related to *S*. *bicolor* in the Poaceae family (S6 Fig). GO analysis of these specific gene families revealed that a majority of them were involved in metabolic processes and biological regulation (Fig 2B and S4 Fig). Gene family classification showed that adlay was closest in relation to sorghum compared with the other two plant species because they shared the most number of gene families. This is consistent with our BLAST results that also showed maximum sequence similarity between adlay and sorghum (S6 Fig). Also, phylogenetic and Ka/Ks analysis using several common transcription factor genes, such as heat shock factor and AP2-EREBP, also confirmed that adlay is evolutionarily closer to sorghum than maize and rice (S10 Fig and S6 Table), which is consistent with other studies based on chloroplast DNA sequences [69, 70]. The identified gene families in this study will be valuable for understanding the biological response mechanism as well as facilitating molecular breeding in adlay.

### Assembly verification using prolamin genes

Adlay is known to contain large amounts of seed-storage proteins known as prolamins [25]. Therefore, we selected prolamin-associated genes from our assembled gene set and compared them with known prolamin genes. From a total of 39 genes that were identified to encode prolamin proteins based on BLAST results (S7 Table), 33 genes contained full length ORFs and six genes had 5’ partial ORFs without a start codon. The identified genes were similar to the previously reported 31 adlay prolamins (https://www.ncbi.nlm.nih.gov/genbank/) in nucleotide length, isoelectric point (pI), and molecular weight (S7 and S8 Tables). Most of the putative prolamin-encoding genes showed high similarity with known adlay prolamin genes at the amino acid sequence level except for three genes (S9 Table).

**Fig 3:**
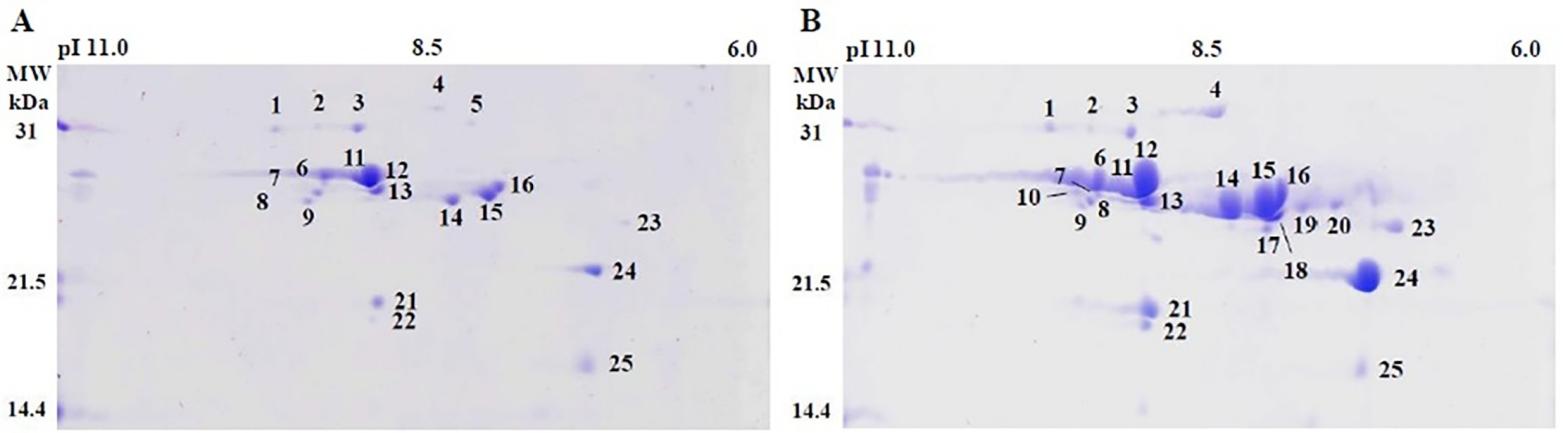
Two-dimensional gel analysis of total prolamin extracts from adlay young (A) and mature (B) seeds.

To analyze the storage protein content during adlay seed development, we performed two-dimensional gel electrophoresis (2-DGE) on young (Fig 3A) and mature seed (Fig 3B) tissues. Two experimental replicates showed that prolamin spots were mainly expressed in the molecular weight range of 14.4–31.0 kDa (Fig 3). We found that 11 spots were two times larger in volume in mature seeds compared with young seeds. Spots 17, 18, 19, and 20 were only seen in mature seeds (Fig 3 and S10 Table). These spots may represent putative prolamins involved in seed development. Prolamins are the major seed storage proteins in maize, sorghum, sugarcane, foxtail millet, and adlay [28, 29]. A recent study has shown that adlay has two main α-prolamin subclasses with molecular weights of 19 and 22 kDa [29]. We predict that spot 21 represents the 19 kDa sub-class and spot 24 potentially represent the 22 kDa α-prolamin subclass. Detailed comparisons of our experimental results with the screened prolamin genes will be conducted in a future study.

**Fig 4:**
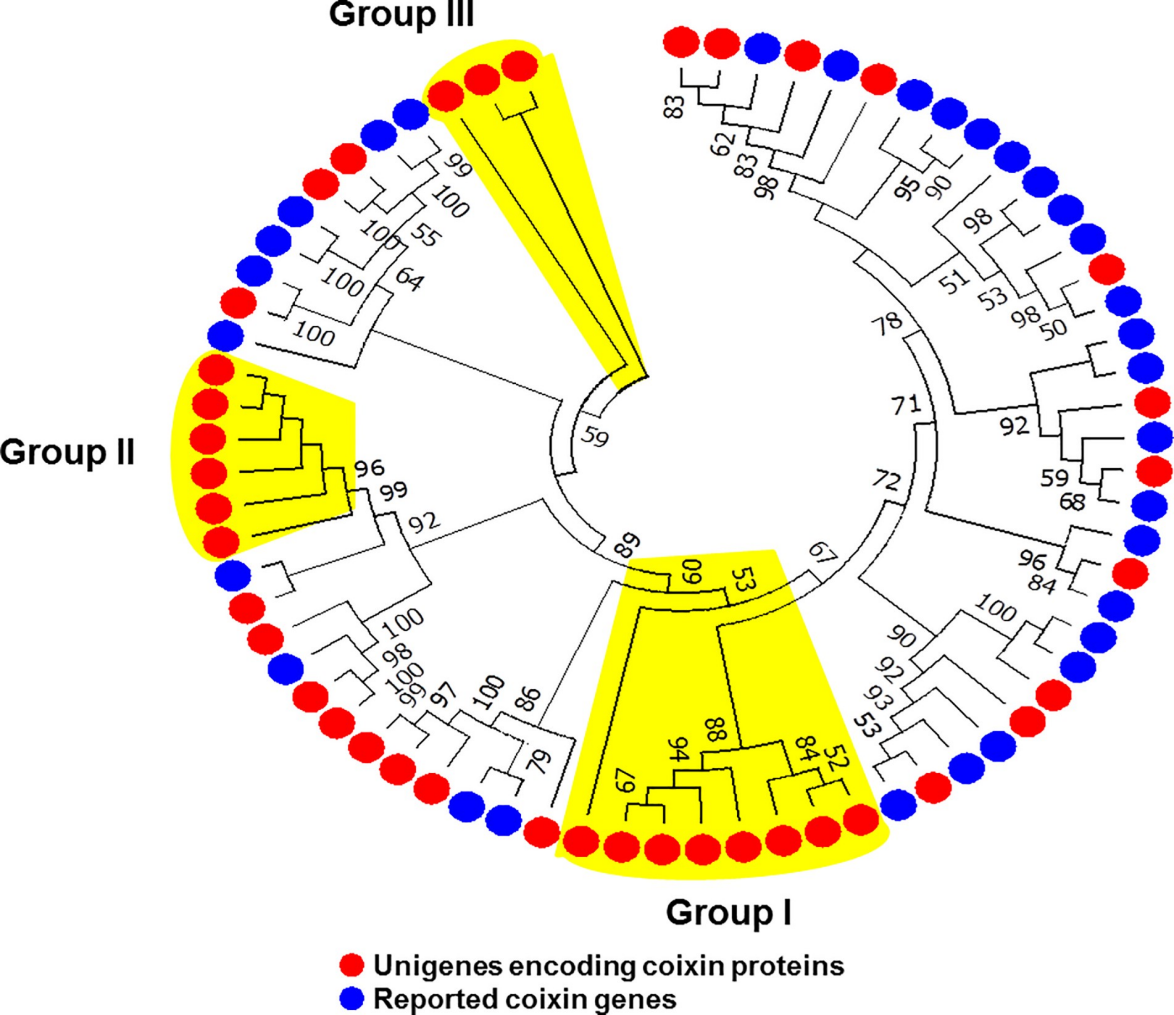
Phylogenetic tree of prolamin-encoding genes. Tree was based on amino acid sequence similarity between predicted genes (red circles) from this study and reported genes (blue circles) in adlay. Gene IDs and gene names are omitted for the sake of legibility (Detailed information can be found in S11 Fig).

Phylogenetic analysis was performed using deduced amino acid sequences of the 39 prolamin-encoding genes from this study with the reported 31 adlay prolamin genes. The phylogenetic tree showed that our prolamin-encoding gene set contained most of the adlay prolamin genes, including both reported and novel genes. A total of 17 prolamin-encoding genes were classified into three groups, Group I to III, which were located separately from other genes (Fig 4 and S11 Fig).

### Assembly verification using vitamin E genes

**Fig 5:**
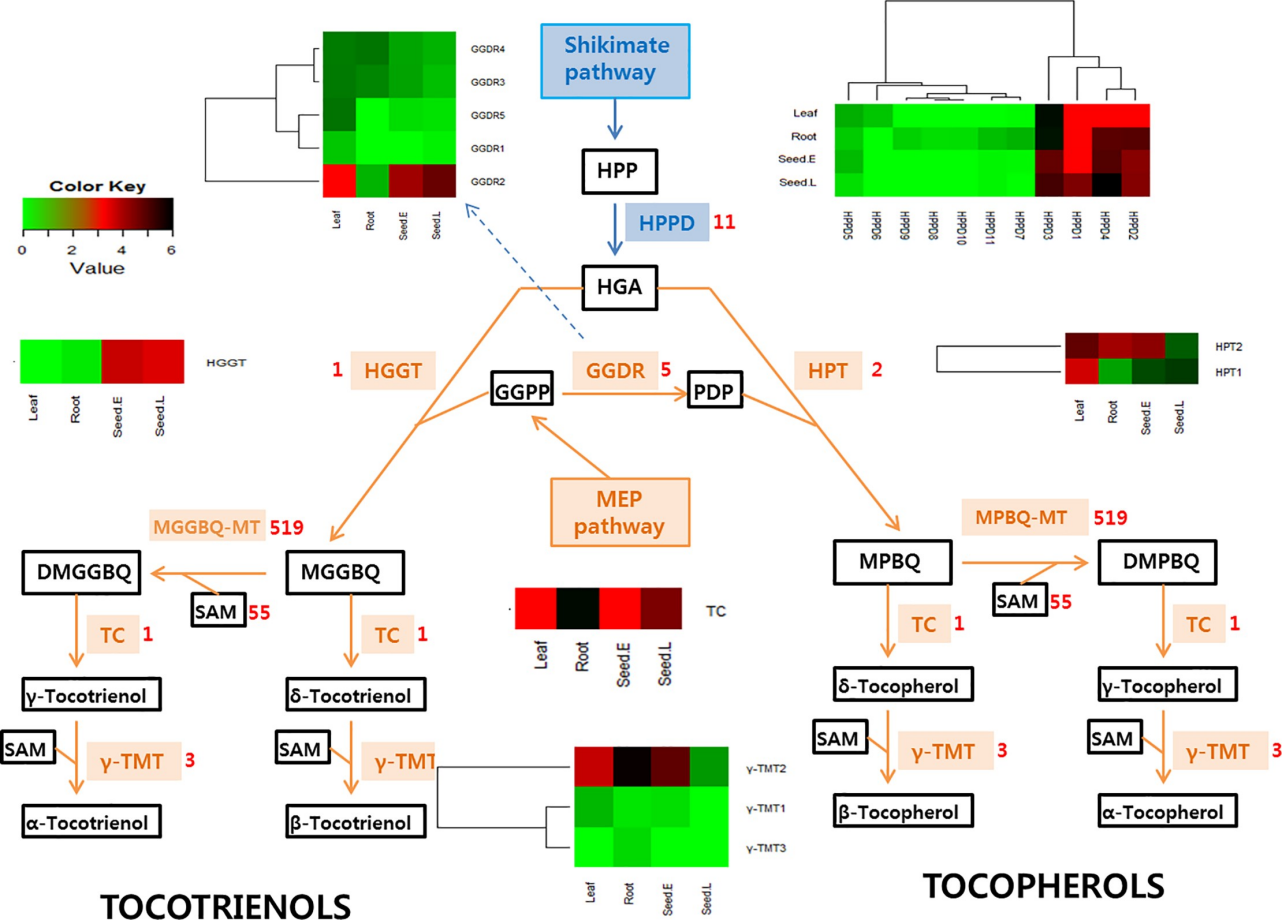
Genes predicted to be involved in the vitamin E synthesis pathway. Vitamin E synthesis is largely divided into two pathways that are regulated by homogentisate geranylgeranyl transferase (HGGT) and homogentisate phytyltransferase (HPT). The 9 differentially expressed enzymes in vitamin E synthesis are ρ-hydroxy phenyl pyruvic acid dioxygenase (HPPD), 2-methyl-6-phytyl benzoquinone methyltransferase (MPBQ-MT), 2-methyl-6-geranylgeranyl-plastoquinol methyltransferase (MGGBQ-MT), tocopherol cyclase (TC), γ-tocopherol methyltransferase (γ-TMT), geranylgeranyl diphosphate reductase (GGDR), homogentisate geranylgeranyl transferase (HGGT), homogentisate phytyltransferase (HPT), and S-adenosylmethionine (SAM). Heat map analysis shows the differential tissue-specific expression of 22 genes associated with the vitamin E pathways. The color bar indicates the value of expression in a sample, based on the color key at the upper left corner. Black indicates upregulation and green, downregulation.

The adlay plant is enriched in various valuable compounds such as vitamin E, squalene, and phytosterols, which may contribute to its nourishing properties [30]. We analyzed the expression patterns of genes that coded for the two main vitamin E subclasses, tocopherols and tocotrienols. Based on FPKM values, we detected 9 differentially-expressed vitamin E pathway enzymes from adlay leaf, root, and seed tissues. BLAST results identified a total of 22 genes associated with the 9 vitamin E pathway enzymes (Fig 5).

Generally, vitamin E consists of eight major isoforms: four saturated tocopherols (α, β, γ, and δ) and four unsaturated tocotrienols (α, β, γ, and δ) and vitamin E synthesis is largely divided into the tocotrienol and tocopherol synthesis pathways. However, we detected six isomers of vitamin E (four tocopherols and two tocotrienols) by gas chromatography-time-of-flight mass spectrometry. Adlay leaf tissues showed the highest amounts of α-, β-, and γ-tocopherols. These results were consistent with the higher levels of homogentisate phytyltransferase (HPT) in leaf tissues compared with those in the other tissues. We detected α-tocotrienol in root and mature seed tissues and γ- tocotrienol in leaf and mature seed tissues. The most prominent vitamin E isoforms in each organ were the α- and γ- tocopherols and tocotrienols, possibly due to high enzyme activity of 2-methyl-6-geranylgeranyl-plastoquinol methyltransferase (MGGBQ-MT) and 2-methyl-6-phytyl benzoquinone methyltransferase (MPBQ-MT) in all tissues (S11 Table). Our results suggested that the adlay leaf and seed tissues were important sources for genes that can potentially be manipulated for the purposes of breeding and genetic engineering.

In conclusion, we performed a *de novo* assembly of the transcriptome of adlay (*C*. *lacryma-jobi*) using full-length Iso-Seq and short-read RNA-Seq. Deep transcriptome analysis generated 57,901 genes from short-read sequencing and 31,177 genes from SMRT long-read sequencing. We validated the assembled gene sets via gene expression analyses, gene family studies, qRT-PCR, and quantitative experiments. We also screened the adlay transcriptome for prolamin- and vitamin E biosynthesis-related genes and performed a comparative analysis of gene families between adlay and other members of the Poaceae family, such as rice, sorghum, and maize. The new adlay gene sequences identified in our study will provide a valuable resource for future genetic and molecular experimentation in adlay.

## Supporting information

S1 Table Unigene-specific primers used for tissue-specific qRT-PCR. (PDF)

S2 Table General properties of the reads produced by short-read sequencing using the Illumina Hiseq 2500 sequencing platform. (PDF)

S3 Table General properties of the reads produced by long-read sequencing using the PacBio sequencing platform. (PDF)

S4 Table Assembly statistics of the genome between RNA-Seq and Iso-Seq. (PDF)

S5 Table Classification of tissue-specific and tissue-enriched genes. (PDF)

S6 Table Analysis of synonymous among adlay, sorghum, maize and rice. (PDF)

S7 Table List of 39 prolamin-encoding genes in this study. (PDF)

S8 Table List of 31 known adlay prolamin genes. (PDF)

S9 Table Amino acid sequence similarity between 39 prolamin-encoding genes and 31 known prolamin (coixin) genes. (PDF)

S10 Table The calculated spot volume values for prolamin storage protein content during seed development (young vs. mature seeds) obtained from 2-DGE. (PDF)

S11 Table Tocopherol and tocotrienol contents in the adlay leaf, root, and young and mature seed tissues. (PDF)

S1 Fig Medicinal plant adlay (*Coix lacryma-jobi*). The leaf, root, young and mature seeds.
(JPG)

S2 Fig Comparison of unigene length distribution between different sequencing platforms. Red and blue bars represent percent of unigenes from Iso-Seq and RNA-Seq, respectively. Y axis is percent of unigenes length distribution.
(JPG)

S3 Fig The number of genes annotated by BLASTX with an E-value threshold of 10^−5^ against different protein databases. The numbers in the circle indicate the number of genes annotated by multiple databases with RNA-Seq (A) and Iso-Seq (B).
(JPG)

S4 Fig Gene ontology (GO) classification of genes from RNA-Seq and Iso-Seq. 27,299 and 31,506 genes from RNA-Seq (green) and Iso-Seq (brown), respectively, were categorized into three functional categories: cellular component, molecular function, and biological process. The GO categories were generated using WEGO (http://wego.genomics.org.cn).
(JPG)

S5 Fig Distribution of transcripts (1,529 for RNA-Seq and 1,209 for Iso-Seq) that encode for transcription factors. (JPG)

S6 Fig Species distribution of the top BLAST hits. Top-hit species from RNA-Seq and Iso-Seq were calculated based on sequence alignments with the lowest E-value obtained from BLAST.
(JPG)

S7 Fig Heatmaps representing the top 10 genes that showed tissue-specific expression in the adlay leaf, root, and young and mature seeds. Red represents high abundance and green represents low abundance.
(JPG)

S8 Fig Heat map analysis of the top 25 genes that were upregulated or downregulated during seed development. Names of genes are indicated on the right. Red represents high abundance and green represents low abundance. To analyze gene expression during seed development, we identified the expressed genes using a filtering log2 (fold change) criterion value of > 1 (corrected p values < 0.001 based on the three replicates).
(JPG)

S9 Fig ClueGO analysis of upregulated and downregulated unigenes during adlay seed development. Identified genes were used as queries to detect homologs against maize proteins. A total of 1,091 (up-regulated) and 2,840 (down-regulated) genes showed significant homology to maize proteins. Over-represented biological processes with upregulated (A) and downregulated (B) genes are shown.
(JPG)

S10 Fig Phylogenetic tree of heat shock factor (HSF) genes. Among 8,747 common gene families, encoding 37 HSF genes were selected and tree represents based on amino acid sequence similarity of HSF genes. Multiple sequence alignments of the amino acid sequences were performed using MUSCLE (MEGA 7 software) and the phylogenetic tree was generated using the Maximum Likelihood (ML) method. Scale bar represents the number of amino acid substitution per site. The bootstrap support values (> 50%) are shown near the branches of the tree. Three classes, A, B and C, of HSFs indicate on the right side of tree. Genes belonging to each of six groups of common gene families are marked by blue boxes on tree. Among 12 HSF genes of adlay, six were more closely located with sorghum HSF genes than those of maize and rice whereas the remaining six were closed to maize or both of sorghum and maize.
(JPG)

S11 Fig Phylogenetic tree of prolamin protein genes. Tree was based on amino acid sequence similarity between predicted genes (red circles) from this study and known adlay genes (blue circles). Multiple sequence alignments of the amino acid sequences were performed using MUSCLE (MEGA 7 software) and the phylogenetic tree was generated using the Neighbor-joining (NJ) method. The bootstrap support values are shown near the branches of the tree. Three groups of prolamin genes, Group I to III, located apart from other genes are indicated.
(JPG)

## References

[1] Otsuka H, Hirai Y, Nagao T, Yamasaki K. Anti-inflammatory activity of benzoxazinoids from roots of Coix lachryma-jobi var. ma-yuen. Journal of Natural Products. 1988;51(1):74–9. 2453615 10.1021/np50055a009

[2] Huang D-W, Chung C-P, Kuo Y-H, Lin Y-L, Chiang W. Identification of compounds in adlay (Coix lachryma-jobi L. var. ma-yuen Stapf) seed hull extracts that inhibit lipopolysaccharide-induced inflammation in RAW 264.7 macrophages. Journal of agricultural and food chemistry. 2009;57(22):10651–7. 10.1021/jf9028514 19886607 10.1021/jf9028514

[3] Huang D-W, Kuo Y-H, Lin F-Y, Lin Y-L, Chiang W. Effect of Adlay (Coix lachryma-jobi L. var. ma-yuen Stapf) Testa and its phenolic components on Cu2+-treated low-density lipoprotein (LDL) oxidation and lipopolysaccharide (LPS)-induced inflammation in RAW 264.7 macrophages. Journal of agricultural and food chemistry. 2009;57(6):2259–66. 10.1021/jf803255p 19243096 10.1021/jf803255p

[4] Chen H-J, Chung C-P, Chiang W, Lin Y-L. Anti-inflammatory effects and chemical study of a flavonoid-enriched fraction from adlay bran. Food Chemistry. 2011;126(4):1741–8. 10.1016/j.foodchem.2010.12.074 25213953 10.1016/j.foodchem.2010.12.074

[5] Chen H-H, Chiang W, Chang J-Y, Chien Y-L, Lee C-K, Liu K-J, et al Antimutagenic constituents of adlay (Coix lachryma-jobi L. var. ma-yuen Stapf) with potential cancer chemopreventive activity. Journal of agricultural and food chemistry. 2011;59(12):6444–52. 10.1021/jf200539r 21561091 10.1021/jf200539r

[6] Numata M, Yamamoto A, Moribayashi A, Yamada H. Antitumor components isolated from the Chinese herbal medicine Coix lachryma-jobi. Planta Medica. 1994;60(04):356–9. 7938271 10.1055/s-2006-959500

[7] Chang H-C, Huang Y-C, Hung W-C. Antiproliferative and chemopreventive effects of adlay seed on lung cancer in vitro and in vivo. Journal of agricultural and food chemistry. 2003;51(12):3656–60. 10.1021/jf021142a 12769541 10.1021/jf021142a

[8] Shih C-K, Chiang W, Kuo M-L. Effects of adlay on azoxymethane-induced colon carcinogenesis in rats. Food and chemical toxicology. 2004;42(8):1339–47. 10.1016/j.fct.2004.03.011 15207385 10.1016/j.fct.2004.03.011

[9] Lee M-Y, Lin H-Y, Cheng F, Chiang W, Kuo Y-H. Isolation and characterization of new lactam compounds that inhibit lung and colon cancer cells from adlay (Coix lachryma-jobi L. var. ma-yuen Stapf) bran. Food and Chemical Toxicology. 2008;46(6):1933–9. 10.1016/j.fct.2008.01.033 18331775 10.1016/j.fct.2008.01.033

[10] Chung C-P, Hsu C-Y, Lin J-H, Kuo Y-H, Chiang W, Lin Y-L. Antiproliferative lactams and spiroenone from adlay bran in human breast cancer cell lines. Journal of agricultural and food chemistry. 2011;59(4):1185–94. 10.1021/jf104088x 21284381 10.1021/jf104088x

[11] Li SC, Chen CM, Lin SH, Chiang W, Shih CK. Effects of adlay bran and its ethanolic extract and residue on preneoplastic lesions of the colon in rats. Journal of the Science of Food and Agriculture. 2011;91(3):547–52. 10.1002/jsfa.4219 21218491 10.1002/jsfa.4219

[12] Qu D, Sun W, Liu M, Liu Y, Zhou J, Chen Y. Bitargeted microemulsions based on coix seed ingredients for enhanced hepatic tumor delivery and synergistic therapy. International journal of pharmaceutics. 2016;503(1–2):90–101. 10.1016/j.ijpharm.2016.03.001 26947738 10.1016/j.ijpharm.2016.03.001

[13] Hsu H-Y, Lin B-F, Lin J-Y, Kuo C-C, Chiang W. Suppression of allergic reactions by dehulled adlay in association with the balance of TH1/TH2 cell responses. Journal of Agricultural and Food Chemistry. 2003;51(13):3763–9. 10.1021/jf021154w 12797741 10.1021/jf021154w

[14] Chen H-J, Shih C-K, Hsu H-Y, Chiang W. Mast cell-dependent allergic responses are inhibited by ethanolic extract of adlay (Coix lachryma-jobi L. var. ma-yuen Stapf) testa. Journal of agricultural and food chemistry. 2010;58(4):2596–601. 10.1021/jf904356q 20102206 10.1021/jf904356q

[15] Chen H-J, Lo Y-C, Chiang W. Inhibitory effects of adlay bran (Coix lachryma-jobi L. var. ma-yuen Stapf) on chemical mediator release and cytokine production in rat basophilic leukemia cells. Journal of ethnopharmacology. 2012;141(1):119–27. 10.1016/j.jep.2012.02.009 22353428 10.1016/j.jep.2012.02.009

[16] Kim SO, Yun S-J, Jung B, Lee EH, Hahm D-H, Shim I, et al Hypolipidemic effects of crude extract of adlay seed (Coix lachrymajobi var. mayuen) in obesity rat fed high fat diet: Relations of TNF-α and leptin mRNA expressions and serum lipid levels. Life sciences. 2004;75(11):1391–404. 10.1016/j.lfs.2004.03.006 15234196 10.1016/j.lfs.2004.03.006

[17] Huang B-W, Chiang M-T, Yao H-T, Chiang W. The effect of adlay oil on plasma lipids, insulin and leptin in rat. Phytomedicine. 2005;12(6–7):433–9. 10.1016/j.phymed.2004.02.010 16008119 10.1016/j.phymed.2004.02.010

[18] Kim SO, Yun S-J, Lee EH. The water extract of adlay seed (Coix lachrymajobi var. mayuen) exhibits anti-obesity effects through neuroendocrine modulation. The American journal of Chinese medicine. 2007;35(02):297–308. 17436369 10.1142/S0192415X07004825

[19] Ha DT, Nam Trung T, Bich Thu N, Van On T, Hai Nam N, Van Men C, et al Adlay seed extract (Coix lachryma-jobi L.) decreased adipocyte differentiation and increased glucose uptake in 3T3-L1 cells. Journal of medicinal food. 2010;13(6):1331–9. 10.1089/jmf.2010.1155 21091246 10.1089/jmf.2010.1155

[20] Ishiguro Y, Okamoto K, Sakamoto H, Sonoda Y. Antimicrobial substances coixindens A and B in etiolated seedlings of adlay [Coix lachryma-jobi]. Journal of the Agricultural Chemical Society of Japan (Japan). 1993.

[21] Kuo C-C, Shih M-C, Kuo Y-H, Chiang W. Antagonism of free-radical-induced damage of adlay seed and its antiproliferative effect in human histolytic lymphoma U937 monocytic cells. Journal of agricultural and food chemistry. 2001;49(3):1564–70. 11312897 10.1021/jf001215v

[22] Yu F, Gao J, Zeng Y, Liu CX. Effects of adlay seed oil on blood lipids and antioxidant capacity in hyperlipidemic rats. Journal of the Science of Food and Agriculture. 2011;91(10):1843–8. 10.1002/jsfa.4393 21452173 10.1002/jsfa.4393

[23] Wang L, Chen J, Xie H, Ju X, Liu RH. Phytochemical profiles and antioxidant activity of adlay varieties. Journal of agricultural and food chemistry. 2013;61(21):5103–13. 10.1021/jf400556s 23647066 10.1021/jf400556s

[24] Wang L, Chen C, Su A, Zhang Y, Yuan J, Ju X. Structural characterization of phenolic compounds and antioxidant activity of the phenolic-rich fraction from defatted adlay (Coix lachryma-jobi L. var. ma-yuen Stapf) seed meal. Food chemistry. 2016;196:509–17. 10.1016/j.foodchem.2015.09.083 26593521 10.1016/j.foodchem.2015.09.083

[25] Ottoboni LM, Leite A, Targon MLN, Crozier A, Arruda P. Characterization of the storage protein in seed of Coix lacryma-jobi var. Adlay. Journal of agricultural and food chemistry. 1990;38(3):631–5.

[26] Leite A, Ottoboni LM, Targon MLP, Silva MJ, Turcinelli SR, Arruda P. Phylogenetic relationship of zeins and coixins as determined by immunological cross-reactivity and Southern blot analysis. Plant molecular biology. 1990;14(5):743–51. 2102852 10.1007/BF00016507

[27] Ottoboni L, Leite A, Yunes J. Sequence analysis of 22 kd-like α-prolamin genes from coix, maize and sorghum reveals a highly conserved protein structure and regulatory elements. Plant Mol Biol. 1993;21:765–78. 8467075 10.1007/BF00027110

[28] Zhou L, Huang B, Meng X, Wang G, Wang F, Xu Z, et al The amplification and evolution of orthologous 22-kDa α-prolamin tandemly arrayed genes in coix, sorghum and maize genomes. Plant molecular biology. 2010;74(6):631–43. 10.1007/s11103-010-9705-5 20938800 10.1007/s11103-010-9705-5

[29] Correa de Souza RS, Balbuena TS, Arruda P. Structure, Organization, and Expression of the Alpha Prolamin Multigenic Family Bring New Insights into the Evolutionary Relationships among Grasses. The Plant Genome. 2015;8(1). 10.3835/plantgenome2014.06.0027 33228278

[30] Wu T-T, Charles AL, Huang T-C. Determination of the contents of the main biochemical compounds of Adlay (Coxi lachrymal-jobi). Food chemistry. 2007;104(4):1509–15.

[31] Qureshi AA, Sami SA, Salser WA, Khan FA. Dose-dependent suppression of serum cholesterol by tocotrienol-rich fraction (TRF25) of rice bran in hypercholesterolemic humans. Atherosclerosis. 2002;161(1):199–207. 11882333 10.1016/s0021-9150(01)00619-0

[32] Yuen KH, Wong JW, Lim AB, Ng BH, Choy WP. Effect of mixed-tocotrienols in hypercholesterolemic subjects. Functional Foods in Health and Disease. 2011;1(3):106–17.

[33] Nesaretnam K, Meganathan P, Veerasenan SD, Selvaduray KR. Tocotrienols and breast cancer: the evidence to date. Genes & nutrition. 2012;7(1):3. 21516480 10.1007/s12263-011-0224-z PMC3250526

[34] Springett GM, Husain K, Neuger A, Centeno B, Chen D-T, Hutchinson TZ, et al A phase I safety, pharmacokinetic, and Pharmacodynamic Presurgical trial of vitamin E δ-tocotrienol in patients with pancreatic ductal neoplasia. EBioMedicine. 2015;2(12):1987–95. 10.1016/j.ebiom.2015.11.025 26844278 10.1016/j.ebiom.2015.11.025 PMC4703733

[35] Kuhad A, Bishnoi M, Tiwari V, Chopra K. Suppression of NF-κβ signaling pathway by tocotrienol can prevent diabetes associated cognitive deficits. Pharmacology Biochemistry and Behavior. 2009;92(2):251–9. 10.1016/j.pbb.2008.12.012 19138703

[36] Kuhad A, Chopra K. Tocotrienol attenuates oxidative–nitrosative stress and inflammatory cascade in experimental model of diabetic neuropathy. Neuropharmacology. 2009;57(4):456–62. 10.1016/j.neuropharm.2009.06.013 19555701 10.1016/j.neuropharm.2009.06.013

[37] Tiwari V, Kuhad A, Bishnoi M, Chopra K. Chronic treatment with tocotrienol, an isoform of vitamin E, prevents intracerebroventricular streptozotocin-induced cognitive impairment and oxidative–nitrosative stress in rats. Pharmacology Biochemistry and Behavior. 2009;93(2):183–9. 10.1016/j.pbb.2009.05.009 19464315

[38] Tiwari V, Kuhad A, Chopra K. Tocotrienol ameliorates behavioral and biochemical alterations in the rat model of alcoholic neuropathy. PAIN. 2009;145(1–2):129–35. 10.1016/j.pain.2009.05.028 19541419 10.1016/j.pain.2009.05.028

[39] Norazlina M, Lee P, Lukman H, Nazrun A, Ima-Nirwana S. Effects of vitamin E supplementation on bone metabolism in nicotine-treated rats. Singapore Medical Journal. 2007;48(3):195 17342286

[40] Hermizi H, Faizah O, Ima-Nirwana S, Nazrun SA, Norazlina M. Beneficial effects of tocotrienol and tocopherol on bone histomorphometric parameters in Sprague–Dawley male rats after nicotine cessation. Calcified Tissue International. 2009;84(1):65–74. 10.1007/s00223-008-9190-x 19020790 10.1007/s00223-008-9190-x

[41] Azlina MN, Nafeeza M, Khalid B. A comparison between tocopherol and tocotrienol effects on gastric parameters in rats exposed to stress. Asia Pacific journal of clinical nutrition. 2005;14(4):358 16326642

[42] Shi X, Zhang C, Liu Q, Zhang Z, Zheng B, Bao M. De novo comparative transcriptome analysis provides new insights into sucrose induced somatic embryogenesis in camphor tree (Cinnamomum camphor a L.). BMC genomics. 2016;17(1):26. 26727885 10.1186/s12864-015-2357-8 PMC4700650

[43] NGS Q. Toolkit: a toolkit for quality control of next generation sequencing data Patel Ravi K.; Jain Mukesh. PLoS One. 2012;7(2):e30619 10.1371/journal.pone.0030619 22312429 10.1371/journal.pone.0030619 PMC3270013

[44] Schmieder R, Lim YW, Edwards R. Identification and removal of ribosomal RNA sequences from metatranscriptomes. Bioinformatics. 2011;28(3):433–5. 10.1093/bioinformatics/btr669 22155869 10.1093/bioinformatics/btr669 PMC3268242

[45] Haas BJ, Papanicolaou A, Yassour M, Grabherr M, Blood PD, Bowden J, et al De novo transcript sequence reconstruction from RNA-seq using the Trinity platform for reference generation and analysis. Nature protocols. 2013;8(8):1494 10.1038/nprot.2013.084 23845962 10.1038/nprot.2013.084 PMC3875132

[46] Li W, Godzik A. Cd-hit: a fast program for clustering and comparing large sets of protein or nucleotide sequences. Bioinformatics. 2006;22(13):1658–9. 10.1093/bioinformatics/btl158 16731699 10.1093/bioinformatics/btl158

[47] Altschul SF, Gish W, Miller W, Myers EW, Lipman DJ. Basic local alignment search tool. Journal of molecular biology. 1990;215(3):403–10. 10.1016/S0022-2836(05)80360-2 2231712 10.1016/S0022-2836(05)80360-2

[48] Conesa A, Götz S. Blast2GO: A comprehensive suite for functional analysis in plant genomics. International journal of plant genomics. 2008;2008. 10.1155/2008/619832 PMC2375974 18483572

[49] Ye J, Fang L, Zheng H, Zhang Y, Chen J, Zhang Z, et al WEGO: a web tool for plotting GO annotations. Nucleic acids research. 2006;34(suppl_2):W293–W7. 16845012 10.1093/nar/gkl031 PMC1538768

[50] Langmead B, Salzberg SL. Fast gapped-read alignment with Bowtie 2. Nature methods. 2012;9(4):357 10.1038/nmeth.1923 22388286 10.1038/nmeth.1923 PMC3322381

[51] Li B, Dewey CN. RSEM: accurate transcript quantification from RNA-Seq data with or without a reference genome. BMC bioinformatics. 2011;12(1):323. 21816040 10.1186/1471-2105-12-323 PMC3163565

[52] Anders S, Huber W. Differential expression analysis for sequence count data. Genome biology. 2010;11(10):R106 10.1186/gb-2010-11-10-r106 20979621 10.1186/gb-2010-11-10-r106 PMC3218662

[53] Bindea G, Mlecnik B, Hackl H, Charoentong P, Tosolini M, Kirilovsky A, et al ClueGO: a Cytoscape plug-in to decipher functionally grouped gene ontology and pathway annotation networks. Bioinformatics. 2009;25(8):1091–3. 10.1093/bioinformatics/btp101 19237447 10.1093/bioinformatics/btp101 PMC2666812

[54] Jin J, Tian F, Yang D-C, Meng Y-Q, Kong L, Luo J, et al PlantTFDB 4.0: toward a central hub for transcription factors and regulatory interactions in plants. Nucleic acids research. 2016:gkw982. 10.1093/nar/gkw982 PMC5210657 27924042

[55] Li L, Stoeckert CJ, Roos DS. OrthoMCL: identification of ortholog groups for eukaryotic genomes. Genome research. 2003;13(9):2178–89. 10.1101/gr.1224503 12952885 10.1101/gr.1224503 PMC403725

[56] Livak KJ, Schmittgen TD. Analysis of relative gene expression data using real-time quantitative PCR and the 2− ΔΔCT method. methods. 2001;25(4):402–8. 10.1006/meth.2001.1262 11846609 10.1006/meth.2001.1262

[57] Bradford MM. A rapid and sensitive method for the quantitation of microgram quantities of protein utilizing the principle of protein-dye binding. Analytical biochemistry. 1976;72(1–2):248–54. 942051 10.1016/0003-2697(76)90527-3

[58] Kim JK, Park S-Y, Na J-K, Seong ES, Yu CY. Metabolite profiling based on lipophilic compounds for quality assessment of perilla (Perilla frutescens) cultivars. Journal of agricultural and food chemistry. 2012;60(9):2257–63. 10.1021/jf204977x 22329700 10.1021/jf204977x

[59] Xu Z, Peters RJ, Weirather J, Luo H, Liao B, Zhang X, et al Full‐length transcriptome sequences and splice variants obtained by a combination of sequencing platforms applied to different root tissues of S alvia miltiorrhiza and tanshinone biosynthesis. The Plant Journal. 2015;82(6):951–61. 10.1111/tpj.12865 25912611 10.1111/tpj.12865

[60] DeLay B, Mamidala P, Wijeratne A, Wijeratne S, Mittapalli O, Wang J, et al Transcriptome analysis of the salivary glands of potato leafhopper, Empoasca fabae. Journal of insect physiology. 2012;58(12):1626–34. 10.1016/j.jinsphys.2012.10.002 23063500 10.1016/j.jinsphys.2012.10.002

[61] Stafford-Banks CA, Rotenberg D, Johnson BR, Whitfield AE, Ullman DE. Analysis of the salivary gland transcriptome of Frankliniella occidentalis. PloS one. 2014;9(4):e94447 10.1371/journal.pone.0094447 24736614 10.1371/journal.pone.0094447 PMC3988053

[62] Fukushima A, Nakamura M, Suzuki H, Yamazaki M, Knoch E, Mori T, et al Comparative characterization of the leaf tissue of physalis alkekengi and physalis peruviana using RNA-seq and metabolite profiling. Frontiers in plant science. 2016;7:1883 10.3389/fpls.2016.01883 28066454 10.3389/fpls.2016.01883 PMC5167740

[63] Liu Y, Wang Y, Guo F, Zhan L, Mohr T, Cheng P, et al Deep sequencing and transcriptome analyses to identify genes involved in secoiridoid biosynthesis in the Tibetan medicinal plant Swertia mussotii. Scientific reports. 2017;7:43108 10.1038/srep43108 28225035 10.1038/srep43108 PMC5320516

[64] Yang CQ, Fang X, Wu XM, Mao YB, Wang LJ, Chen XY. Transcriptional Regulation of Plant Secondary Metabolism F. Journal of integrative plant biology. 2012;54(10):703–12. 10.1111/j.1744-7909.2012.01161.x 22947222 10.1111/j.1744-7909.2012.01161.x

[65] Cai Z, Liu H, He Q, Pu M, Chen J, Lai J, et al Differential genome evolution and speciation of Coix lacryma-jobi L. and Coix aquatica Roxb. hybrid guangxi revealed by repetitive sequence analysis and fine karyotyping. BMC genomics. 2014;15(1):1025. 25425126 10.1186/1471-2164-15-1025 PMC4256728

[66] Chakrabortee S, Boschetti C, Walton LJ, Sarkar S, Rubinsztein DC, Tunnacliffe A. Hydrophilic protein associated with desiccation tolerance exhibits broad protein stabilization function. Proceedings of the National Academy of Sciences. 2007;104(46):18073–8. 10.1073/pnas.0706964104 PMC2084298 17984052

[67] Charng Y-y, Liu H-c, Liu N-y, Hsu F-c, Ko S-s. Arabidopsis Hsa32, a novel heat shock protein, is essential for acquired thermotolerance during long recovery after acclimation. Plant Physiology. 2006;140(4):1297–305. 10.1104/pp.105.074898 16500991 10.1104/pp.105.074898 PMC1435801

[68] Liu X, Fu J, Gu D, Liu W, Liu T, Peng Y, et al Genome-wide analysis of gene expression profiles during the kernel development of maize (Zea mays L.). Genomics. 2008;91(4):378–87. 10.1016/j.ygeno.2007.12.002 18280698 10.1016/j.ygeno.2007.12.002

[69] Leseberg CH, Duvall MR. The complete chloroplast genome of Coix lacryma-jobi and a comparative molecular evolutionary analysis of plastomes in cereals. Journal of molecular evolution. 2009;69(4):311–8. 10.1007/s00239-009-9275-9 19777151 10.1007/s00239-009-9275-9

[70] Teerawatananon A, Jacobs SW, Hodkinson TR. Phylogenetics of Panicoideae (Poaceae) based on chloroplast and nuclear DNA sequences. Telopea. 2011;13(1–2):115–42.

