# Supplementary material for: De novo transcriptome assembly of the Chinese pearl barley, adlay, by full-length isoform and short-read RNA sequencing
Source: PLoS One. 2018 Dec 11;13(12):e0208344. doi: 10.1371/journal.pone.0208344 (PMC6289447; doi:10.1371/journal.pone.0208344)
Supplement: S1 Table — (PDF) [file pone.0208344.s001.pdf]

S1 Table. Unigene-specific primers used for tissue-specific qRT-PCR.

Annotation	Unigene ID	Primer sequence (5’→3’)	Amplicon (bp)
Fructose-bisphosphate aldolase, chloroplastic	c179643_g1F<br>c179643_g1R	CAGGATGCCCTCAAACATC<br>AATTGTGGAGCCCGAAATC	130
Phylloplanin -like	c179599_g1F<br>c179599_g1R	GATGTGTGCCACAACGTGTA<br>ATCAGCACACAGCAATCTGG	106
Photosystem I subunit O	c47839_g2F<br>c47839_g2R	CTTGCAGCGGATGAAATAGA<br>CCAGCTCCAGAGTGTAGCAA	111
Probable O-methyltransferase 2	c37561_g1F<br>c37561_g1R	AGTTGCTCCTGCTTTCCAAG<br>ACATGATGTACCACCGTCCA	135
14 kDa proline-rich protein DC2.15	c70641_g2F<br>c70641_g2R	GCAAGCACACGTACGGAGTA<br>CCGGATTCATGTGCCTCTA	145
Aquaporin TIP2-2	c138525_g1F<br>c138525_g1R	CGCACGAGATCTTTACATCG<br>GTCGTCGTTTGCTTTGCTC	149
Non-specific lipid	c97785_g1F<br>c97785_g1R	GAGGCAGGCACATCATCTC<br>CAACGTCAACTGCAACACG	150
Thioredoxin H-type	c158944_g1F<br>c158944_g1R	CGATGAACTCCTTGATGGTG<br>CGAACTGGACGAAGTTGCTA	137
Non-specific lipid -transfer protein 2	c64755_g1F<br>c64755_g1R	TGCGTACGTGGTGGAAATTA<br>GCAGATCGATGTCCTACGTG	149
Late embryogenesis abundant protein	c536_g1F<br>c536_g1R	TACGAAGCGTCAAACACACC<br>AGTCGTCAAAGTCGCAGGTT	137
Defensin-like protein 2	c99521_g1F<br>c99521_g1R	GTGATGGAAACGTTGCTGAG<br>ACCATGCATGTAAGGTGACG	126
1-Cys peroxiredoxin PER1	c28148_g1F<br>c28148_g1R	TCTCGAACTCCTTGGCGTA<br>GATCCGCATCCACGACTAC	122
Transcription factor BTF3(Internal control)	c5718_g1F<br>c5718_g1R	GTCGTCATCATCGTCCTGAG<br>CTTGGTCCTGACAACTTGGA	123
