# Supplementary material for: De novo transcriptome assembly of the Chinese pearl barley, adlay, by full-length isoform and short-read RNA sequencing
Source: PLoS One. 2018 Dec 11;13(12):e0208344. doi: 10.1371/journal.pone.0208344 (PMC6289447; doi:10.1371/journal.pone.0208344)
Supplement: S2 Table — (PDF) [file pone.0208344.s002.pdf]

S2 Table. General properties of the reads produced by short-read sequencing using the Illumina Hiseq 2500 sequencing platform.

Hiseq sequencing library	Hiseq2500 reads	High-quality reads rate (>=Q30)	Mapping rate
Leaf-1	19303203	91.25%	78.85%
Leaf-2	21569456	91.21%	78.92%
Leaf-3	21500707	91.08%	78.95%
Root-1	17892913	91.00%	78.70%
Root-2	17683001	91.10%	75.16%
Root-3	19020852	91.01%	78.42%
Young seed-1	20309644	91.54%	75.61%
Young seed-2	19777262	90.62%	78.48%
Young seed-3	19775010	91.31%	78.58%
Mature seed-1	18650087	93.76%	64.78%
Mature seed-2	17372508	93.32%	65.93%
Mature seed-3	17720343	93.50%	68.05%
