# Supplementary material for: De novo transcriptome assembly of the Chinese pearl barley, adlay, by full-length isoform and short-read RNA sequencing
Source: PLoS One. 2018 Dec 11;13(12):e0208344. doi: 10.1371/journal.pone.0208344 (PMC6289447; doi:10.1371/journal.pone.0208344)
Supplement: S3 Table — (PDF) [file pone.0208344.s003.pdf]

S3 Table. General properties of the reads produced by long-read sequencing using the PacBio sequencing platform.

PacBio sequencing library	PacBio reads	Full-length reads	High-quality isoforms
1-2 kb	157,874	95,628	39,792
2-3 kb	172,762	92,071	28,624
3-6 kb	282,771	147,324	42,229
