# Supplementary material for: De novo transcriptome assembly of the Chinese pearl barley, adlay, by full-length isoform and short-read RNA sequencing
Source: PLoS One. 2018 Dec 11;13(12):e0208344. doi: 10.1371/journal.pone.0208344 (PMC6289447; doi:10.1371/journal.pone.0208344)
Supplement: S4 Table — (PDF) [file pone.0208344.s004.pdf]

**S4 Table. Assembly statistics of the genome between RNA-Seq and Iso-Seq.**

| Source Assembler | RNA-Seq Trinity | Iso-Seq |
|---|---|---|
| Number of unigens | 111,850 | 31,177 |
| Total size (bp) | 96,864,135 | 77,155,540 |
| Minimum length (bp) | 300 | 340 |
| Maximum length (bp) | 16,649 | 5,620 |
| Average length (bp) | 866 | 2,475 |
| N50 length (bp) | 1,220 | 2,999 |
| GC content (%) | 47.96 | 48.19 |
