# Supplementary material for: De novo transcriptome assembly of the Chinese pearl barley, adlay, by full-length isoform and short-read RNA sequencing
Source: PLoS One. 2018 Dec 11;13(12):e0208344. doi: 10.1371/journal.pone.0208344 (PMC6289447; doi:10.1371/journal.pone.0208344)
Supplement: S5 Table — (PDF) [file pone.0208344.s005.pdf]

S5 Table. Classification of tissue-specific and tissue-enriched genes.

Category	Leaf	Root	Young seed	Mature seed	Total
Tissue-specific	810	832	169	374	2,185
Tissue-enriched	375	283	138	243	1,039
