# Supplementary material for: De novo transcriptome assembly of the Chinese pearl barley, adlay, by full-length isoform and short-read RNA sequencing
Source: PLoS One. 2018 Dec 11;13(12):e0208344. doi: 10.1371/journal.pone.0208344 (PMC6289447; doi:10.1371/journal.pone.0208344)
Supplement: S6 Table — (PDF) [file pone.0208344.s006.pdf]

**S6 Table. Analysis of synonymous among adlay, sorghum, maize and rice.**

| Group [a] | Cl-Sb | | | Cl-Zm | | | Cl-Os | | |
|---|---|---|---|---|---|---|---|---|---|
| | Ka [b] | Ks | Ka/Ks | Ka | Ks | Ka/Ks | Ka | Ks | Ka/Ks |
| HSF [c] | 0.034 | 0.178 | 0.234 | 0.052 | 0.269 | 0.252 | 0.118 | 0.874 | 0.157 |
| AP2 | 0.058 | 0.251 | 0.246 | 0.129 | 0.468 | 0.253 | 0.193 | 1.010 | 0.198 |
| Total | 0.051 | 0.228 | 0.242 | 0.105 | 0.407 | 0.252 | 0.170 | 0.968 | 0.185 |

| Group | Sb-Zm | | | Sb-Os | | | Zm-Os | | |
|---|---|---|---|---|---|---|---|---|---|
| | Ka | Ks | Ka/Ks | Ka | Ks | Ka/Ks | Ka | Ks | Ka/Ks |
| HSF | 0.050 | 0.310 | 0.196 | 0.103 | 0.802 | 0.144 | 0.127 | 0.899 | 0.152 |
| AP2 | 0.116 | 0.412 | 0.261 | 0.211 | 1.132 | 0.204 | 0.209 | 1.127 | 0.188 |
| Total | 0.096 | 0.381 | 0.241 | 0.177 | 1.031 | 0.185 | 0.184 | 1.057 | 0.177 |

[a] Cl : adlay, Sb : sorghum, Zm : maize, Os : rice.

[b]Ka : Non-synonymous substitution, Ks : synonymous substitution, The values were calculated with protein coding gene using PAML or PAL2NAL program.

[c]HSF : heat shock factor, AP2 : AP2-EREBP
