# Supplementary material for: De novo transcriptome assembly of the Chinese pearl barley, adlay, by full-length isoform and short-read RNA sequencing
Source: PLoS One. 2018 Dec 11;13(12):e0208344. doi: 10.1371/journal.pone.0208344 (PMC6289447; doi:10.1371/journal.pone.0208344)
Supplement: S7 Table — (PDF) [file pone.0208344.s007.pdf]

S7 Table. List of 39 prolamin-encoding genes in this study.

ID	Description	Length (nt)[a]	Length (aa)[b]	PI[c]	kDa
c1204_f1p10_904	alpha-coixin 6	904	222	9.0	24.3
c2597_f1p0_860	22 kDa alpha coixin 1	860	166	10.3	17.8
c11544_f1p0_905	22-kD coixin	905	248	9.2	27.1
c11020_f1p0_958	22 kDa alpha coixin 4	958	246	9.5	26.4
c3113_f2p1_657	delta-coixin	657	211	6.3	22.9
c11393_f1p0_770	alpha-coixin 6	770	181	9.1	19.7
c11578_f1p0_698	alpha-coixin	698	181	9.7	19.8
c3401_f1p0_795	gamma-coixin 22KDa	795	206	9.8	20.3
c11930_f1p0_897	22 kDa alpha coixin 4	897	245	8.9	26.4
c2064_f1p1_980	22-kD coixin	980	101	8.0	10.8
c4503_f1p3_1049	22-kD coixin	1049	252	7.0	26.3
c11994_f1p0_891	22 kDa alpha coixin 4	891	245	8.9	26.3
c6621_f6p37_967	alpha-coixin 5	967	271	9.3	29.3
c10758_f1p2_961	22 kDa alpha coixin 6	961	216	9.3	23.1
c209501_f1p0_1058	alpha-coixin 5	1058	160	9.0	17.5
c7985_f1p32_1311	alpha-coixin 17 kDA	1311	195	7.4	20.4
c9078_f1p10_1218	alpha-coixin 17 kDA	1218	195	7.4	20.4
c132764_f1p10_996	alpha-coixin	996	266	9.3	28.9
c133016_f1p18_1732	alpha-coixin 6	1732	241	9.0	26.6
c133162_f1p0_1360	19 kDa alpha coixin-like	1360	99	8.3	11.0
c20756_f1p90_1805	22-kD coixin	1805	293	3.8	42.9
c69190_f1p12_1110	alpha-coixin 7	1110	99	8.3	11.0
c23362_f1p17_1122	alpha-coixin 8	1122	242	8.4	26.6
c156909_f5p32_1244	alpha-coixin 5	1244	163	9.2	17.9
c24952_f1p20_1124	22 kDa alpha coixin 1	1124	312	9.0	33.6
c203871_f1p0_1370	19 kDa alpha coixin-like	1370	99	8.3	11.0
c43937_f1p18_990	22-kD coixin	990	265	9.3	28.8
c195973_f1p0_1455	alpha-coixin 7	1455	119	8.4	12.7
c204131_f1p0_1307	alpha-coixin 7	1307	99	8.3	11.0
c208114_f1p0_1057	alpha-coixin 5	1057	253	8.9	26.8
c211055_f1p0_1230	alpha-coixin 7	1230	99	8.3	11.0
c88105_f1p12_977	alpha-coixin 27 kDA	977	180	9.0	18.9
c117538_f1p2_1193	gamma-coixin	1193	322	6.7	37.6
c126593_f1p4_1590	alpha-coixin 3	1590	159	10.2	17.1
c126933_f2p17_1012	alpha-coixin	1012	308	10.3	33.9
c131028_f1p11_1141	alpha-coixin 7	1141	291	9.2	32.2
c23579_f1p20_2480	alpha-coixin 8	2480	242	8.4	26.6
c9169_f1p24_4012	alpha-coixin 8	4012	242	8.4	26.6
c10582_f1p24_4352	alpha-coixin 6	4352	197	7.9	21.5

[a] Length (NCBI nt), [b] Length (amino acids), [c] Isoelectric point (pI)
