# Supplementary material for: De novo transcriptome assembly of the Chinese pearl barley, adlay, by full-length isoform and short-read RNA sequencing
Source: PLoS One. 2018 Dec 11;13(12):e0208344. doi: 10.1371/journal.pone.0208344 (PMC6289447; doi:10.1371/journal.pone.0208344)
Supplement: S8 Table — (PDF) [file pone.0208344.s008.pdf]

S8 Table. List of 31 known adlay prolamin genes

No.	GenBank .	Gene name	L(aa) [a]	pI [b]	kDa
1	AHB39732	alpha coixin 7	197	7.9	21.5
2	AHB39731	alpha coixin 6	265	9.3	28.6
3	AHB39730	alpha coixin 5	265	8.9	28.7
4	AHB39729	alpha coixin 4	265	7.9	28.6
5	AHB39728	alpha coixin 3	264	8.7	28.5
6	AHB39727	alpha coixin 1	265	8.9	28.6
7	AHB39726	alpha coixin 2-like	313	9.3	33.7
8	AHB39725	alpha coixin 1	313	9.3	33.8
9	ADA71329	coixin	313	9.3	33.7
10	ADA71328	coixin	312	9.0	33.6
11	ADA71327	coixin	264	8.7	28.5
12	ADA71326	coixin	265	7.9	28.6
13	ADA71325	coixin	265	8.9	28.7
14	ADA71324	coixin	266	9.3	28.9
15	ADA71323	coixin	265	8.9	28.6
16	1808330A	gamma coixin	199	8.6	21.4
17	CAA44827	alpha-coixin	266	9.3	29.0
18	CAA40965	alpha-coixin	264	7.9	28.5
19	CAA42513	gamma-coixin	199	8.6	21.4
20	CAA40966	alpha-coixin	194	7.4	20.3
21	ACN58179	delta-coixin	166	8.3	18.0
22	ACN58178	alpha-coixin 8	242	8.4	26.6
23	ACN58177	alpha-coixin 7	291	9.2	32.3
24	ACN58176	alpha-coixin 6	197	7.9	21.5
25	ACN58175	alpha-coixin 5	313	9.3	33.7
26	ACN58174	alpha-coixin 4	266	9.3	28.8
27	ACN58173	alpha-coixin 3	265	9.4	28.5
28	ACN58172	alpha-coixin 2	265	7.9	28.7
29	ACN58171	alpha-coixin 1	264	8.7	28.5
30	ACN58170	gamma-coixin	322	6.7	37.6
31	CAA56257	beta-coixin	194	7.4	20.3

[a] Length (amino acids), [b] Isoelectric point (pI)
