# Supplementary material for: De novo transcriptome assembly of the Chinese pearl barley, adlay, by full-length isoform and short-read RNA sequencing
Source: PLoS One. 2018 Dec 11;13(12):e0208344. doi: 10.1371/journal.pone.0208344 (PMC6289447; doi:10.1371/journal.pone.0208344)
Supplement: S9 Table — (PDF) [file pone.0208344.s009.pdf]

S9 Table. Amino acid sequence similarity between 39 prolamin-encoding genes and 31 known prolamin (coixin) genes.

Encoded Prolamin ID	Reported prolamin [a]	Similarity [b]	Description
c132764_f1p10_996	ADA71324	**100**	
c24952_f1p20_1124	ADA71328	**100**	
c43937_f1p18_990	ADA71324	98.8	
c117538_f1p2_1193	ACN58170	98.7	
c131028_f1p11_1141	ACN58177	96.5	
c10582_f1p24_4352	AHB39732, ACN58176	96.4	
c7985_f1p32_1311	CAA40966, CAA56257	94.8	
c9078_f1p10_1218	CAA40966, CAA56257	94.8	
c23362_f1p17_1122	ACN58178	93.8	
c9169_f1p24_4012	ACN58178	93.8	
c11544_f1p0_905	AHB39730, ADA71325	93.5	
c11930_f1p0_897	ADA71326	92.0	
c11994_f1p0_891	ADA71326	92.0	
c126933_f2p17_1012	ADA71324	86.3	
c1204_f1p10_904	AHB39732, ACN58176	85.6	
c6621_f6p37_967	ACN58173	84.1	
c10758_f1p2_961	AHB39731	81.5	
c133016_f1p18_1732	AHB39732, ACN58176	78.9	
c11020_f1p0_958	CAA40965, ACN58172	77.1	
c208114_f1p0_1057	ACN58173	70.6	
c23579_f1p20_2480	ACN58178	68.2	
c11578_f1p0_698	ADA71324	68.0	
c11393_f1p0_770	AHB39732, ACN58176	66.6	
c4503_f1p3_1049	ACN58173	59.2	
c88105_f1p12_977	CAA40965	56.2	
c126593_f1p4_1590	ACN58176	47.6	
c2597_f1p0_860	ACN58176	45.6	
c209501_f1p0_1058	ADA71329, ACN58175	44.4	
c156909_f5p32_1244	AHB39729, ADA71326, CAA40965, ACN58173, ACN58172	40.3	
c2064_f1p1_980	AHB39730, ADA71325	36.6	
c133162_f1p0_1360	ACN58177	31.2	
c203871_f1p0_1370	ACN58177	31.2	
c204131_f1p0_1307	ACN58177	31.2	
c211055_f1p0_1230	ACN58177	31.2	
c69190_f1p12_1110	ACN58177	31.2	
c3401_f1p0_795	AHB39732, AHB39731, ACN58176	**12.9**	Hypothetical
c195973_f1p0_1455	AHB39732	**10.4**	Hypothetical
c3113_f2p1_657	AHB39732, ACN58176	**8.7**	Zein protein

[a] NCBI/GenBank Accession number

[b] Similarity : Sequence identity matrix of the deduced amino acid sequences of 39 genes and the amino acid sequences of known 31 adlay prolamin (coixin) genes. Multiple alignments of the amino acid sequences were performed using MUSCLE (MEGA 7 software) and the identity matrix was calculated using BioEdit ver. 7.2.5.
