# Supplementary material for: De novo transcriptome assembly of the Chinese pearl barley, adlay, by full-length isoform and short-read RNA sequencing
Source: PLoS One. 2018 Dec 11;13(12):e0208344. doi: 10.1371/journal.pone.0208344 (PMC6289447; doi:10.1371/journal.pone.0208344)
Supplement: S10 Table — (PDF) [file pone.0208344.s010.pdf]

S10 Table. The calculated spot volume values for prolamin storage protein content during seed development (young vs. mature seeds) obtained from 2-DGE.

Spot no.	Young seed [a]	Mature seed [a]	Mature/Young
1	254.4	763.5	3.01
2	162.9	258.3	1.58
3	1158.1	1084.9	0.93
4	142.7	1616.2	11.32
5	204.5	-	-
6	1221.4	2812.2	2.30
7	424.8	722.1	1.67
8	548.4	548.3	0.99
9	365.7	886.6	2.42
10	-	539.5	-
11	1393.8	3823.1	2.74
12	6739.2	11072.4	1.64
13	2460.3	2064.9	0.84
14	2428.1	8942.7	3.68
15	2851.7	9738.7	3.41
16	1844.0	5443.5	2.95
17	-	1996.1	-
18	-	553.0	-
19	-	1183.1	-
20	-	1475.2	-
21	1962.2	2592.4	1.32
22	180.5	2409.6	13.34
23	180.7	1671.9	9.25
24	3255.9	12345.3	3.79
25	886.9	811.9	0.91
total	28667.1	75356.3	2.62

[a] The volume measured at each stage is the average of the two replicate experiments. The spot volume was determined by an Image Master Platinum 7.0
