# Supplementary material for: De novo transcriptome assembly of the Chinese pearl barley, adlay, by full-length isoform and short-read RNA sequencing
Source: PLoS One. 2018 Dec 11;13(12):e0208344. doi: 10.1371/journal.pone.0208344 (PMC6289447; doi:10.1371/journal.pone.0208344)
Supplement: S11 Table — (PDF) [file pone.0208344.s011.pdf]

S11 Table. Tocopherol and tocotrienol contents in the adlay leaf, root, and young and mature seed tissues

**Tissue**	**Tocopherol** (T, μg mg^{-1})				**Tocotrienol** (T3, μg mg^{-1})			
	Alpha-T	Beta-T	Gamma -T	Delta-T	Alpha -T3	Beta-T3	Gamma -T3	Delta -T3
Leaf	172.8 [a]	5.68	14.88	0.14	ND	ND	0.56	ND
Root	1.69	0.19	1.19	ND	0.6	ND	ND	ND
Young seed	10.84	0.82	24.12	0.64	ND	ND	ND	ND
Mature seed	6.36	0.82	9.25	0.52	0.25	ND	1.1	ND

[a] Values are mean of three replicate experiments. Mean values are significantly different from each other at $P < 0.05$ by Duncan's multiple range tests. Group clustering was not indicated because the mean values of the tissue factors were significantly different.
