# Supplementary figures and images for: De novo transcriptome assembly of the Chinese pearl barley, adlay, by full-length isoform and short-read RNA sequencing
Source: PLoS One. 2018 Dec 11;13(12):e0208344. doi: 10.1371/journal.pone.0208344 (PMC6289447; doi:10.1371/journal.pone.0208344)

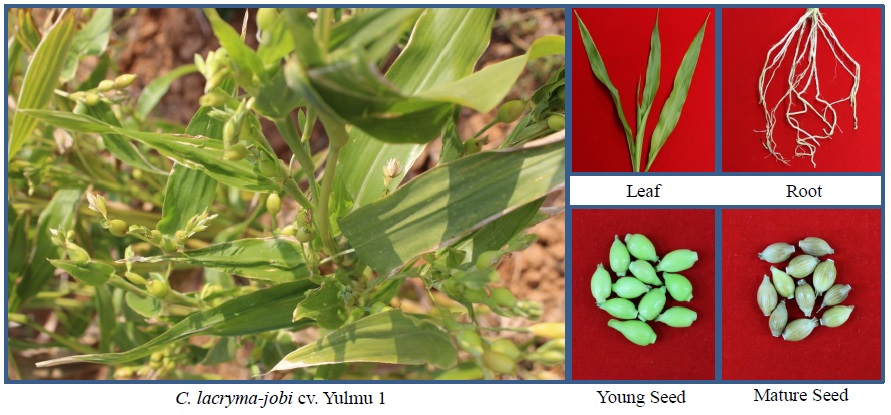

Supplement: S1 Fig — The leaf, root, young and mature seeds. (JPG) [file pone.0208344.s012.jpg]

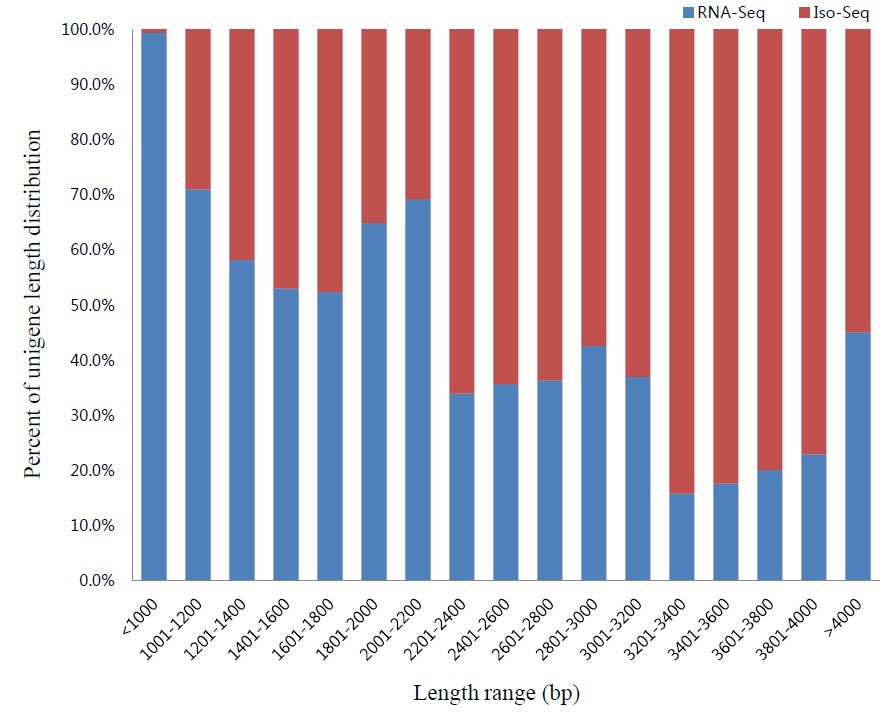

Supplement: S2 Fig — Red and blue bars represent percent of unigenes from Iso-Seq and RNA-Seq, respectively. Y axis is percent of unigenes length distribution. (JPG) [file pone.0208344.s013.jpg]

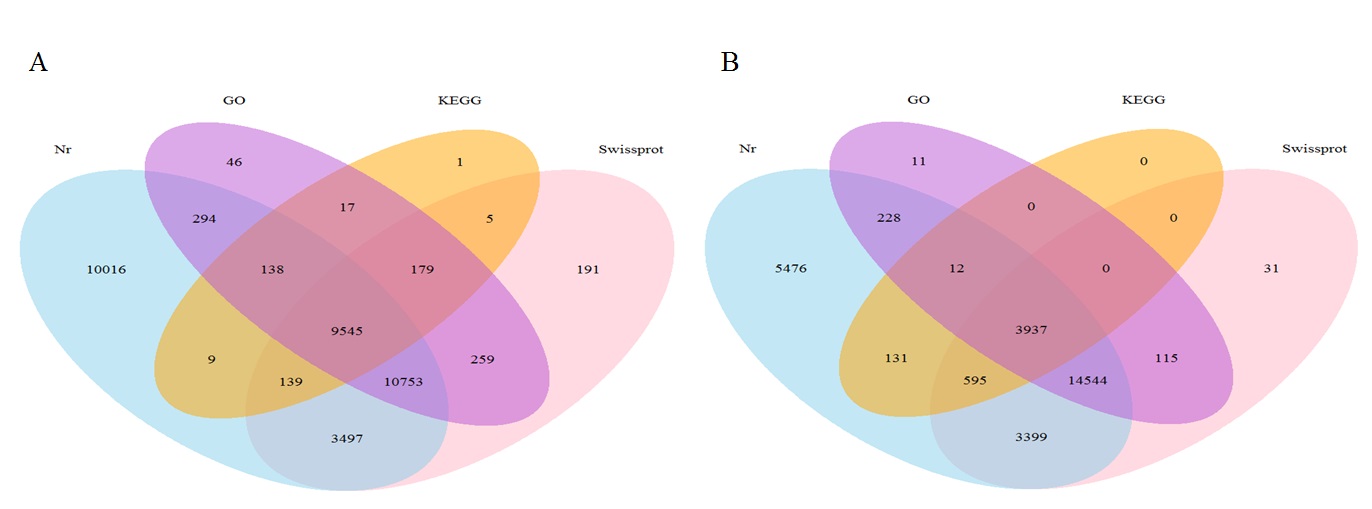

Supplement: S3 Fig — The numbers in the circle indicate the number of genes annotated by multiple databases with RNA-Seq (A) and Iso-Seq (B). (JPG) [file pone.0208344.s014.jpg]

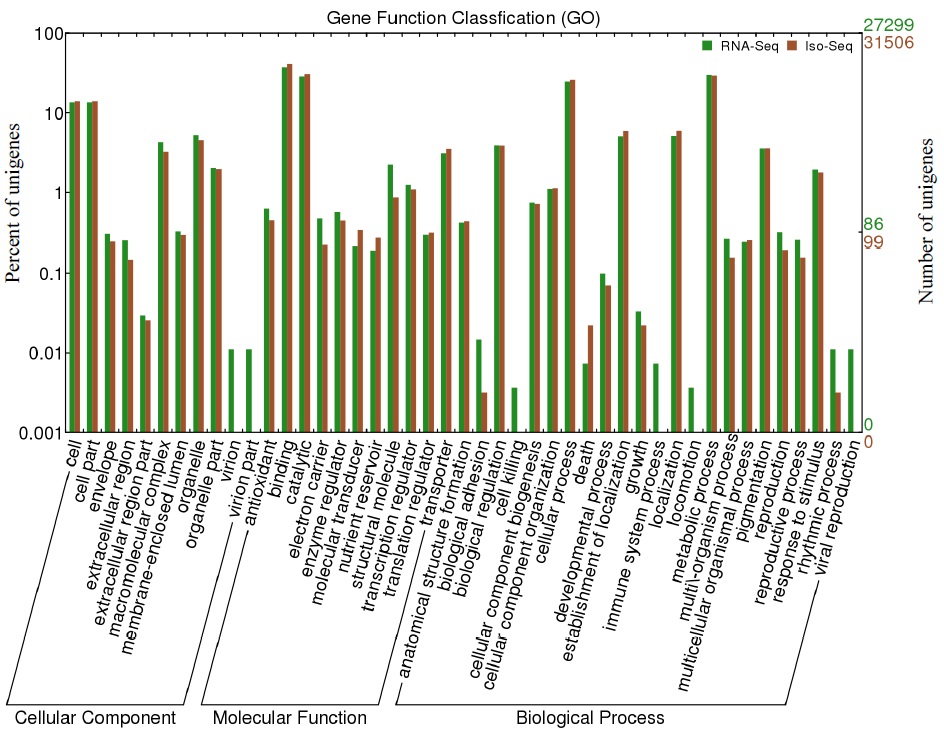

Supplement: S4 Fig — 27,299 and 31,506 genes from RNA-Seq (green) and Iso-Seq (brown), respectively, were categorized into three functional categories: cellular component, molecular function, and biological process. The GO categories were generated using WEGO (http://wego.genomics.org.cn). (JPG) [file pone.0208344.s015.jpg]

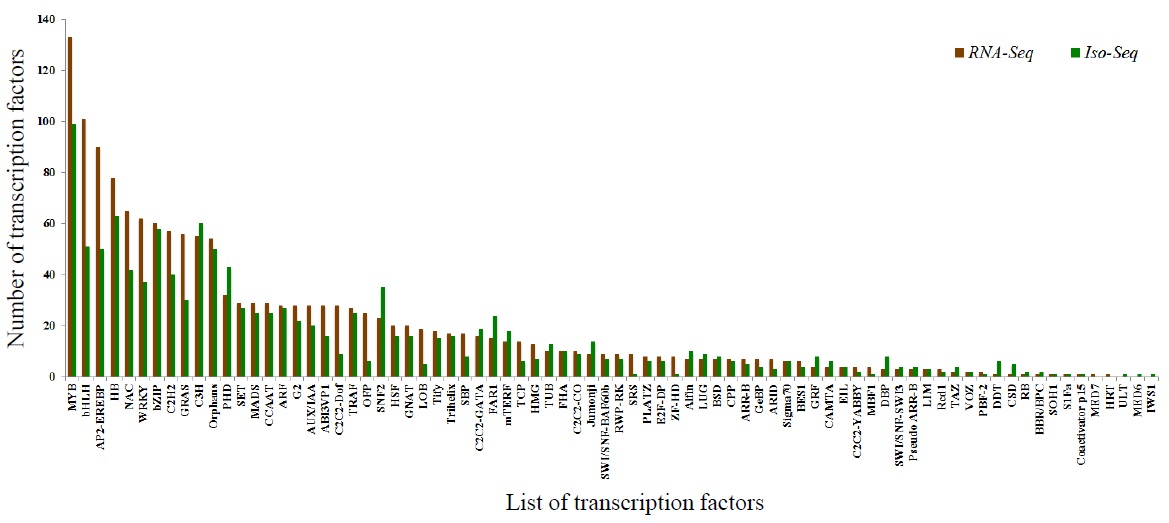

Supplement: S5 Fig — (JPG) [file pone.0208344.s016.jpg]

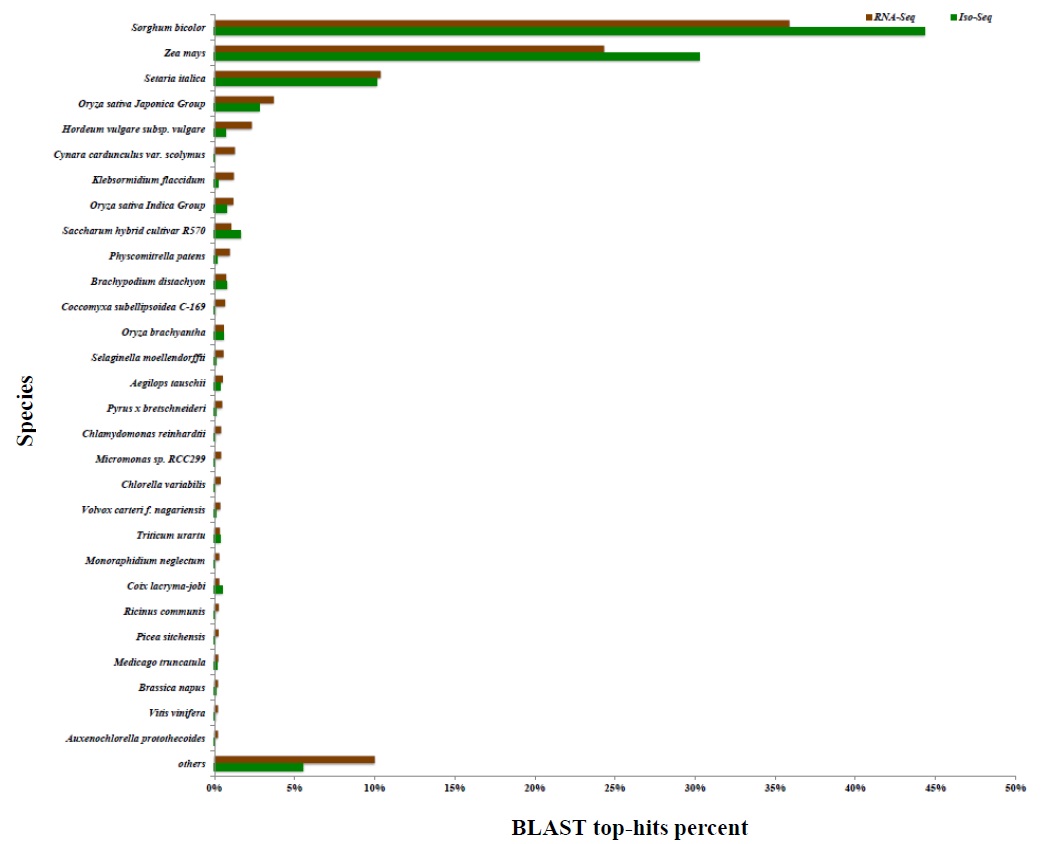

Supplement: S6 Fig — Top-hit species from RNA-Seq and Iso-Seq were calculated based on sequence alignments with the lowest E-value obtained from BLAST. (JPG) [file pone.0208344.s017.jpg]

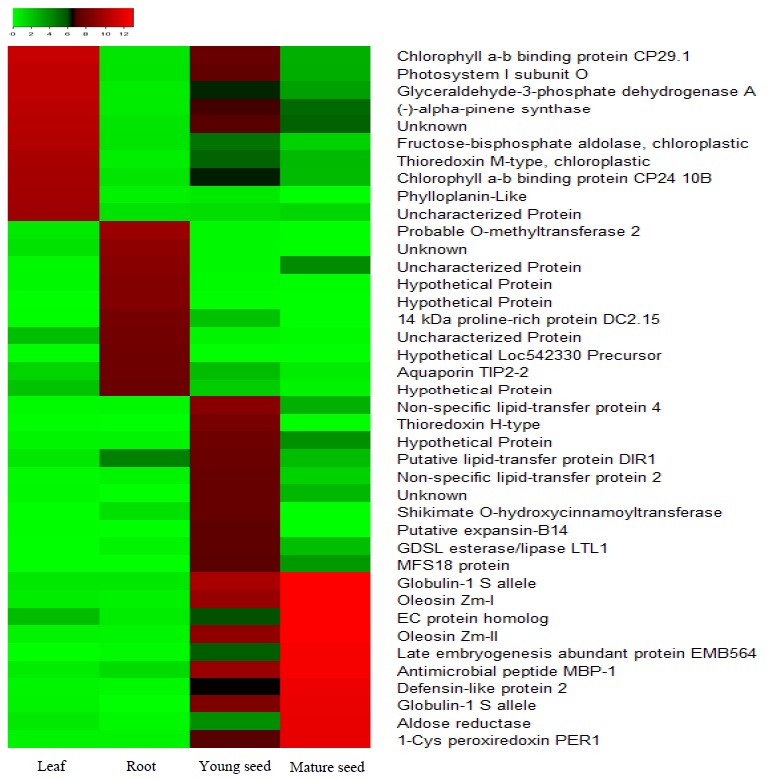

Supplement: S7 Fig — Red represents high abundance and green represents low abundance. (JPG) [file pone.0208344.s018.jpg]

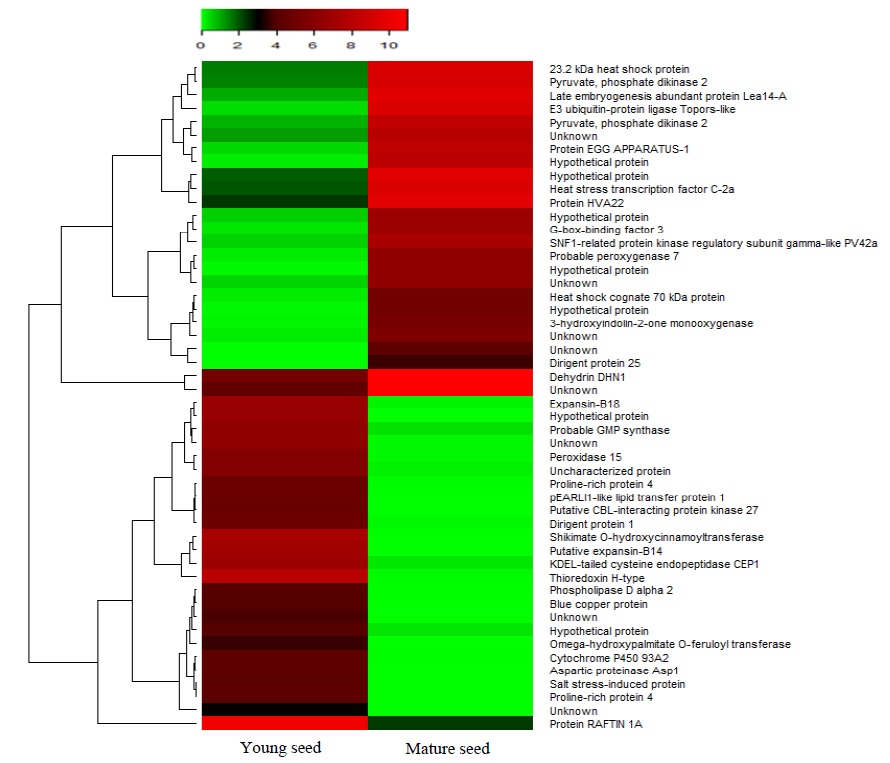

Supplement: S8 Fig — Names of genes are indicated on the right. Red represents high abundance and green represents low abundance. To analyze gene expression during seed development, we identified the expressed genes using a filtering log2 (fold change) criterion value of > 1 (corrected p values < 0.001 based on the three replicates). (JPG) [file pone.0208344.s019.jpg]

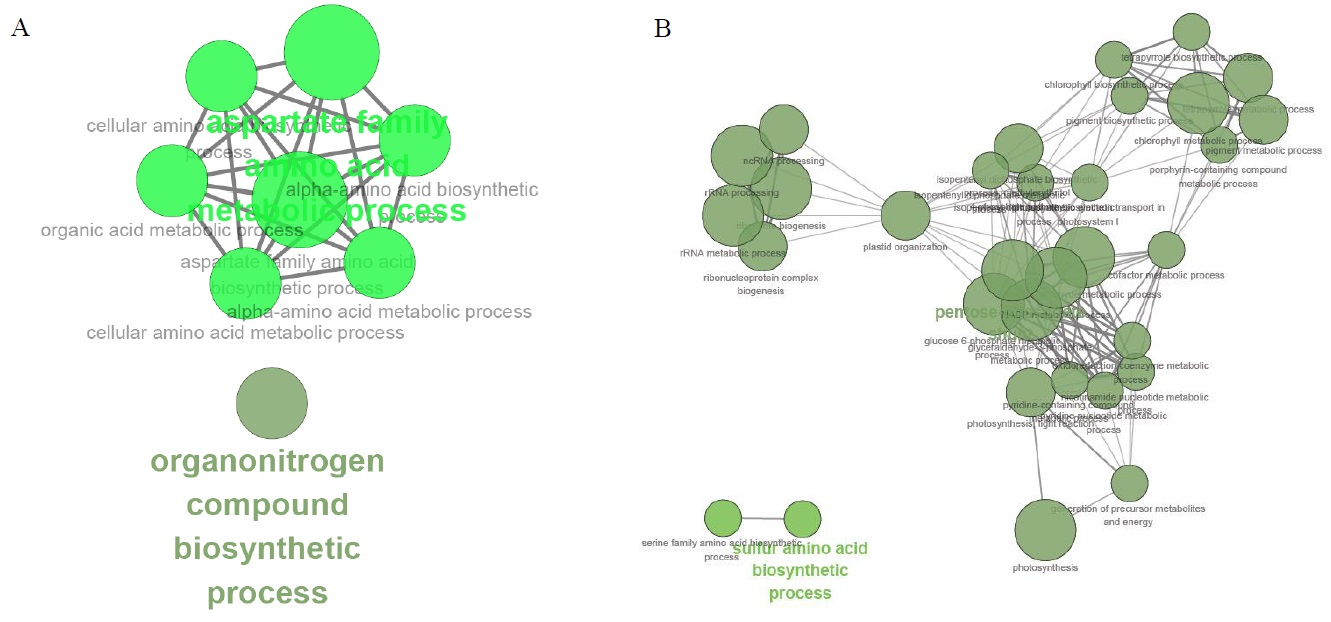

Supplement: S9 Fig — Identified genes were used as queries to detect homologs against maize proteins. A total of 1,091 (up-regulated) and 2,840 (down-regulated) genes showed significant homology to maize proteins. Over-represented biological processes with upregulated (A) and downregulated (B) genes are shown. (JPG) [file pone.0208344.s020.jpg]

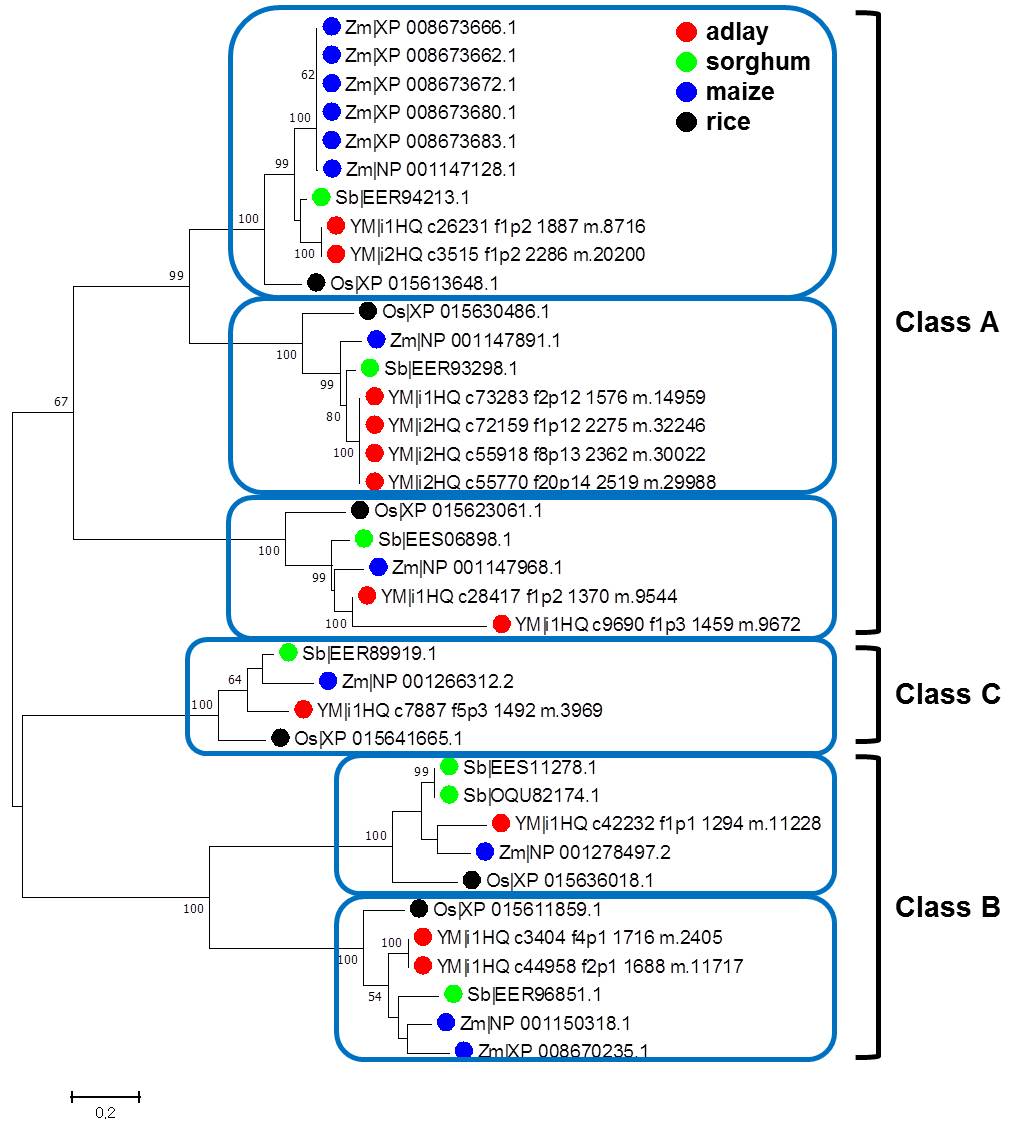

Supplement: S10 Fig — Among 8,747 common gene families, encoding 37 HSF genes were selected and tree represents based on amino acid sequence similarity of HSF genes. Multiple sequence alignments of the amino acid sequences were performed using MUSCLE (MEGA 7 software) and the phylogenetic tree was generated using the Maximum Likelihood (ML) method. Scale bar represents the number of amino acid substitution per site. The bootstrap support values (> 50%) are shown near the branches of the tree. Three classes, A, B and C, of HSFs indicate on the right side of tree. Genes belonging to each of six groups of common gene families are marked by blue boxes on tree. Among 12 HSF genes of adlay, six were more closely located with sorghum HSF genes than those of maize and rice whereas the remaining six were closed to maize or both of sorghum and maize. (JPG) [file pone.0208344.s021.jpg]

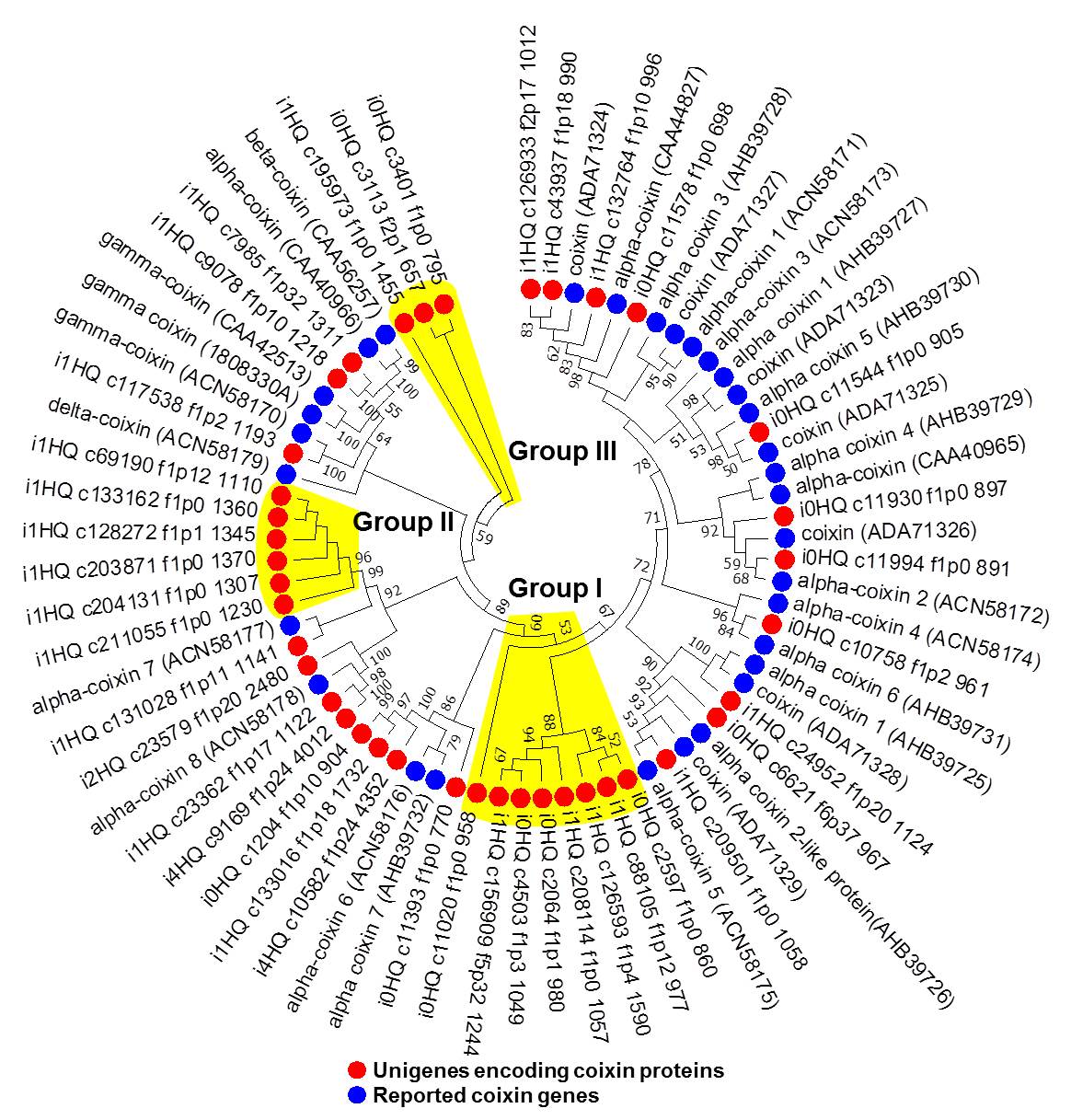

Supplement: S11 Fig — Tree was based on amino acid sequence similarity between predicted genes (red circles) from this study and known adlay genes (blue circles). Multiple sequence alignments of the amino acid sequences were performed using MUSCLE (MEGA 7 software) and the phylogenetic tree was generated using the Neighbor-joining (NJ) method. The bootstrap support values are shown near the branches of the tree. Three groups of prolamin genes, Group I to III, located apart from other genes are indicated. (JPG) [file pone.0208344.s022.jpg]
